# SETD8 inhibits apoptosis and ferroptosis of Ewing’s sarcoma through YBX1/RAC3 axis

**DOI:** 10.1038/s41419-024-06882-5

**Published:** 2024-07-10

**Authors:** Huimou Chen, Jing Hu, Xilin Xiong, Hongling Chen, Qiaofang Liao, Biaojun Lin, Yusong Chen, Yanting Peng, Yang Li, Di Cheng, Zhihua Li

**Affiliations:** 1 Department of Oncology, Sun Yat-sen Memorial Hospital of Sun Yat-sen University, No. 107 Yanjiang Road, Guangzhou, 510120 China; 2 Department of Clinical Laboratory, The Sixth Affiliated Hospital of Sun Yat-sen University, Guangzhou, China; 3 Biomedical Innovation Center, The Sixth Affiliated Hospital of Sun Yat-sen University, Guangzhou, China; 4 Department of Oncology, Medical Centre of Pediatric, Sun Yat-sen Memorial Hospital, Sun Yat-sen University, No. 107 Yanjiang Road, Guangzhou, 510120 China; 5 Department of Clinical Laboratory, Maoming People’s Hospital, Maoming, Guangdong 525000 China; 6 Department of Oncology, Huizhou First Hospital, Huizhou, Guangdong 516000 China

**Keywords:** Paediatric cancer, Targeted therapies

## Abstract

Ewing’s sarcoma (ES) represents a rare yet exceedingly aggressive neoplasm that poses a significant health risk to the pediatric and adolescent population. The clinical outcomes for individuals with relapsed or refractory ES are notably adverse, primarily attributed to the constrained therapeutic alternatives available. Despite significant advancements in the field, molecular pathology-driven therapeutic strategies have yet to achieve a definitive reduction in the mortality rates associated with ES. Consequently, there exists an imperative need to discover innovative therapeutic targets to effectively combat ES. To reveal the mechanism of the SETD8 (also known as lysine methyltransferase 5A) inhibitor UNC0379, cell death manners were analyzed with different inhibitors. The contributions of SETD8 to the processes of apoptosis and ferroptosis in ES cells were evaluated employing the histone methyltransferase inhibitor UNC0379 in conjunction with RNA interference techniques. The molecular regulatory mechanisms of SETD8 in ES were examined through the application of RNA sequencing (RNA-seq) and mass spectrometry-based proteomic analysis. Moreover, nude mouse xenograft models were established to explore the role of SETD8 in ES in vivo. SETD8, a sole nucleosome-specific methyltransferase that catalyzes mono-methylation of histone H4 at lysine 20 (H4K20me1), was found to be upregulated in ES, and its overexpression was associated with dismal outcomes of patients. SETD8 knockdown dramatically induced the apoptosis and ferroptosis of ES cells in vitro and suppressed tumorigenesis in vivo. Mechanistic investigations revealed that SETD8 facilitated the nuclear translocation of YBX1 through post-transcriptional regulatory mechanisms, which subsequently culminated in the transcriptional upregulation of *RAC3*. In summary, SETD8 inhibits the apoptosis and ferroptosis of ES cells through the YBX1/RAC3 axis, which provides new insights into the mechanism of tumorigenesis of ES. SETD8 may be a potential target for clinical intervention in ES patients.

## Introduction

Ewing’s sarcoma (ES) is an infrequent yet highly aggressive solid neoplasm that poses a significant health risk to the pediatric and adolescent population [1]. ES originates most frequently from the bone, and less than 10% of ES arises in the soft tissues. The integration of multimodality therapeutic approaches has significantly enhanced the survival outcomes for patients with limited-stage ES. However, the high risk of relapse and metastasis, and the poor prognosis of patients with ES have led to renewed interest in novel treatment [2]. Approximately 85% of ES cases exhibit chromosomal translocations that lead to the generation of genomic rearrangements involving the breakpoint region 1 (EWSR1) gene and members of the E26 transformation-specific (ETS) family of transcription factors, with the fusion of EWSR1 to FLI1 being the most frequently observed, resulting in the formation of the EWS/FLI fusion protein [3]. This fusion protein, functioning as a deregulated transcription factor or post-transcriptional modulator, is pivotal in the oncogenesis and perpetuation of the sarcomatous state [4]. However, the development of direct inhibitors of EWS/FLI has always been particularly challenging [3]. Consequently, the investigation of additional molecular pathways implicated in the pathogenesis of ES is essential for the advancement of novel and efficacious therapeutic strategies. The development of intrinsic or acquired resistance to apoptotic mechanisms is a critical factor contributing to the failure of therapeutic interventions [5]. As such, it is imperative to devise strategies aiming at either circumventing apoptosis resistance or initiating alternative, non-apoptotic forms of programmed cell death in ES. A substantial body of research has implicated ferroptosis, a form of regulated cell death distinct from apoptosis and characterized by the accumulation of lipid reactive oxygen species (ROS), in the pathobiology of a multitude of human cancers, including breast cancer [6], rhabdomyosarcoma [7], liver cancer [8] and lung cancer [9]. Moreover, a growing corpus of evidence has underscored the potential of ferroptosis induction to augment the therapeutic efficacy of cancer treatment, potentially even overcoming resistance to conventional therapies [5, 10, 11]. Hence, inducing ferroptosis may be a promising potential strategy for cancer therapy.

SETD8, a lysine methyltransferase containing a SET domain that has been extensively investigated, is a sole nucleosome-specific methyltransferase that catalyzes mono-methylation of histone H4 at lysine 20 (H4K20me1) [12]. SETD8 has been characterized as an oncogene in multiple malignancies, exerting its oncogenic effects through the modulation of methylation patterns on target genes. SETD8 contributes to high-risk neuroblastoma growth and apoptosis resistance by silencing p53 [13]; SETD8 promotes the carcinogenesis of colorectal cancer via deregulating the expression of PCNA [14]; SETD8 also represses the ferroptosis of pancreatic cancer cells by downregulating RRAD [15]. However, the role of SETD8 in ES remains unknown.

In the present investigation, we report for the first time the marked overexpression of SETD8 in ES tissues, which correlates with an adverse prognosis in patients afflicted with this disease. Our findings demonstrate that the targeted suppression of SETD8 leads to the induction of both apoptotic and ferroptotic cell deaths in ES cell lines. These results not only underscore the pivotal role of SETD8 in the neoplastic progression of ES but also highlight its potential as a novel therapeutic target for this aggressive cancer.

## Results

### Anticancer activity of UNC0379 and SETD8 knockdown in ES cell lines

**Fig. 1:**
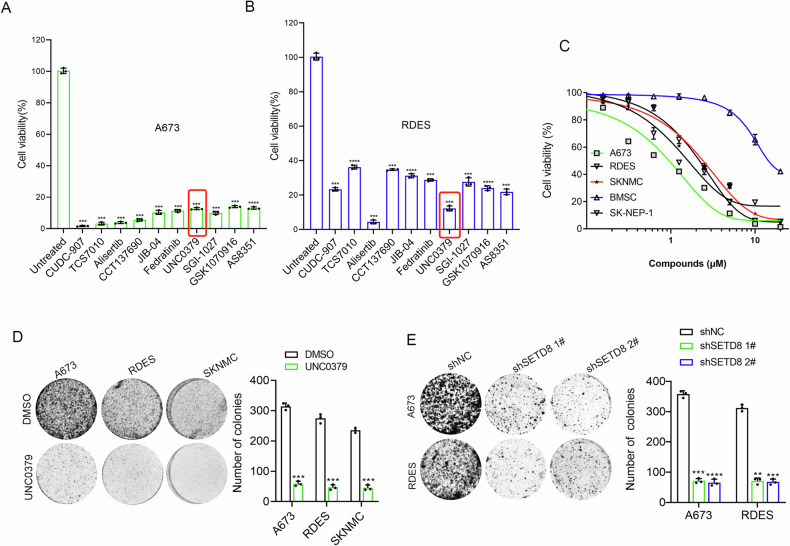
UNC0379 was identified as an effective anticancer agent in ES cells. **A**, **B** The viability of ES cells, specifically the A673 and RDES cell lines, was assessed following treatment with the top 10 anticancer compounds. **C** Bone marrow stem cells (BMSC) served as a normal control group. Both ES and normal BMSC cells were exposed to UNC0379 at the indicated concentration for 48 h, after which cell viability was measured. **D** The colony formation assay demonstrated a significant reduction in the ability of A673, RDES, and SKNMC cells to form colonies after treatment with UNC0379 at 2 μM, as compared to the DMSO control group. **E** The impact of SETD8 knockdown using short hairpin RNA (shRNA) on cell proliferation was evaluated through a colony formation assay. **p < 0.01, ***p < 0.001, ****p < 0.0001.

A systematic screen was conducted on a curated panel of 294 small-molecule compounds sourced from a commercial library. The cytotoxic efficiency of the compounds against A673 cells was quantitatively evaluated using the CCK-8 assay, with the cells being incubated with the respective compounds for a period of 72 h prior to assessment. At a concentration of 2 μM, A673 is sensitive to 31 compounds, which include Aurora kinases, histone methyltransferases or demethylases, HDAC inhibitors, and PARP inhibitors (Fig. S1). The following top 10 compounds were identified: CUDC-907 (a dual HDAC-PI3K inhibitor), TCS7010 (an AURKA inhibitor), Alisertib (an AURKA inhibitor), CCT137690 (an aurora kinase inhibitor), JIB-04 (a dual HDAC-PI3K inhibitor), Fedratinib (a JAK2 inhibitor), UNC0379 (a SETD8 inhibitor), SG1-1027 (DNA methyltransferase inhibitor II), GSK1070916 (Aurora B/C inhibitor), and AS8351 (a KDM5B inhibitor). After that, the efficiency of the above 10 compounds was re-validated in another ES cells RDES (Fig. 1A, B). Due to the accumulating evidence on the antitumor effect in multiple tumors [16, 17], but an unexplored role in ES, UNC0379 was chosen as the objective of interest for further study.

Apart from A673 (IC_50_: 0.81 μM) and RDES (IC_50_: 1.76 μM), the tumor-killing effect of UNC0379 was also verified in other human ES cell lines (SKNMC, IC_50_: 2.2 μM), SK-NEP-1(IC_50_: 2.06 μM). Nevertheless, UNC0379 showed negligible effect on the normal BMSC, indicating its specifical targeting on tumor cells rather than the non-tumor cells BMSC (IC_50_: 15.92 μM) (Fig. 1C). The results of colony formation assays revealed that UNC0379 or SETD8 knockdown significantly compromised the proliferative capabilities of ES cells (Fig. 1D, E). These findings indicated that UNC0379 or SETD8 knockdown has anticancer activity against human ES cells.

### SETD8 was upregulated in ES and associated with poor prognosis of patients, inhibiting apoptosis and ferroptosis of ES cells

**Fig. 2:**
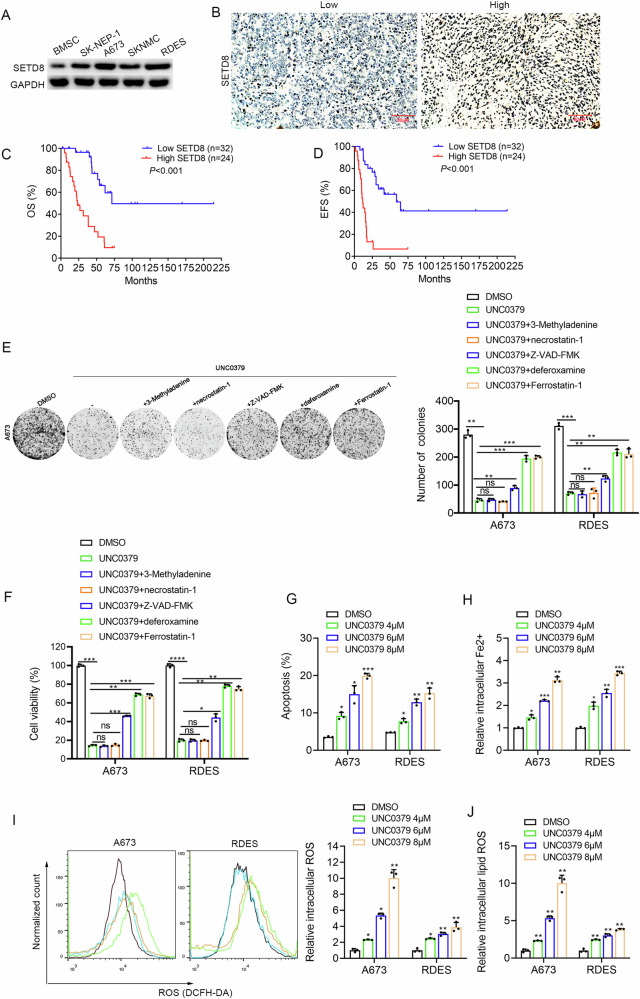
SETD8 was upregulated in ES and associated with poor prognosis of patients, inhibiting apoptosis and ferroptosis of ES cells. **A** WB analysis was conducted to assess the expression levels of the SETD8 protein in BMSC and across four ES cell lines. The original blots corresponding to these experiments are displayed in Fig. S10A. **B** IHC provided representative images of SETD8 protein expression in ES patient samples at a magnification of ×200. **C**, **D** Kaplan–Meier plots depicted the overall survival (OS) and event-free survival (EFS) for ES patients with high versus low SETD8 expression levels. **E** The impact of UNC0379 (2 μM) on the clonogenic capacity of ES cells, in the context of various cell death inhibitors (10 μM Z-VAD-FMK, necrostatin-1, Ferrostatin-1, deferoxamine, 5 mM 3-Methyladenine and 200 μM Methyladenine), was evaluated through a colony formation assay. **F** The viability of A673 and RDES cells following 48 h of exposure to UNC0379 (2 μM) with or without the aforementioned cell death inhibitors was measured using the CCK-8 assay. **G** A column chart illustrated the variation in apoptosis rates among the ES cell lines following SETD8 inhibition by UNC0379. **H** The relative levels of intracellular iron (Fe2+) in ES cells were measured after SETD8 was inhibited by UNC0379. **I** The levels of reactive oxygen species (ROS) within ES cells were determined post-SETD8 inhibition by UNC0379. **J** The lipid ROS levels in ES cells were assessed after SETD8 inhibition by UNC0379. Data are presented as the mean ± standard deviation from three separate experiments. ‘ns’ denotes no statistical significance. *p < 0.05, **p < 0.01, ***p < 0.001, ****p < 0.0001.

As expected, the expression levels of SETD8 were observed to be markedly elevated in ES cell lines when compared with BMSC (Fig. 2A). Subsequently, the clinical relevance of SETD8 in ES was evaluated through immunohistochemistry (IHC) assays, and the representative images were acquired (Fig. 2B). The survival analysis conducted in this study revealed a significant correlation between elevated SETD8 expression levels and the reduced OS and EFS of patients diagnosed with ES (Fig. 2C, D).

To reveal the underlying mechanism after inhibiting SETD8 with UNC0379, the cell death manners were analyzed with different inhibitors, including the cell autophagy inhibitor 3-Methyladenine, the cell necrosis inhibitor necrostatin-1, apoptosis inhibitor Z-VAD-FMK, and ferroptosis inhibitors deferoxamine and Ferrostatin-1. The investigation revealed that cotreatment with UNC0379 and either 3-Methyladenine or necrostatin-1 did not result in a notable alteration in cell proliferation and viability, indicating that UNC0379 is incapable of inducing necrosis and autophagy in ES. Nonetheless, the impact of UNC0379 on cell proliferation and viability was found to be partially mitigated by the apoptosis inhibitor Z-VAD-FMK and the ferroptosis inhibitors deferoxamine and Ferrostatin-1 (Fig. 2E, F). Moreover, the simultaneous application of the apoptosis inhibitor Z-VAD-FMK in conjunction with the ferroptosis inhibitor Ferrostatin-1 has been demonstrated to significantly ameliorate cell death triggered by UNC0379, exhibiting a more pronounced effect than the use of each inhibitor individually. What is worth noting is that the combined use of these two inhibitors cannot completely reverse the cell death induced by UNC0379 (Fig. S2A), which suggests that apoptosis and ferroptosis are two main death manners induced by UNC0379 in ES, with other types of death or influencing factors existing as well. As expected, flow cytometric analysis demonstrated that treatment with UNC0379 led to a pronounced induction of apoptosis in ES, which was observed to be in a dose-dependent fashion (Fig. 2G). Accordingly, the accumulation of iron level (Fig. 2H), the intracellular ROS, and lipid ROS (Fig. 2I, J) were also verified to increase dramatically in a dose-dependent manner, which confirmed the ferroptosis induced by UNC0379.

**Fig. 3:**
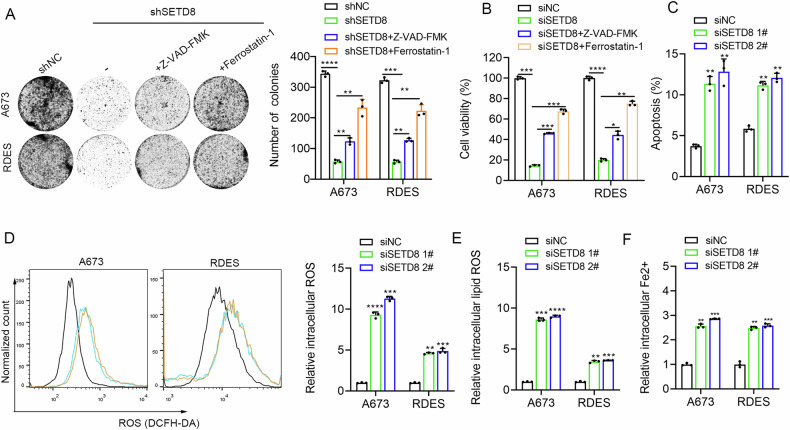
SETD8 knockdown induced apoptosis and ferroptosis of ES cells. **A** The clonogenic potential of SETD8 knockdown in ES cells was evaluated in the presence or absence of specific cell death inhibitors, including 10 μM Z-VAD-FMK, and 10 μM Ferrostatin-1, using a colony formation assay. **B** A673 and RDES cells underwent SETD8 knockdown and were treated with the aforementioned inhibitors for 48 h, after which their viability was measured using the CCK-8 assay. **C** A column chart displayed the shifts in apoptosis rates among the ES cell lines subsequent to SETD8 knockdown. **D** The levels of intracellular ROS in ES cells were quantified following SETD8 knockdown. **E** Intracellular lipid ROS levels were similarly measured in ES cells post-SETD8 knockdown. **F** Relative changes in intracellular Fe2+ levels were observed in ES cells after SETD8 knockdown. The data are expressed as the mean ± standard deviation derived from three individual experiments. *p < 0.05, **p < 0.01, ***p < 0.001, ****p < 0.0001.

Similarly, the effect of SETD8 inhibition through genetic knockdown was found to be partially reversible by the apoptosis inhibitor or ferroptosis inhibitor (Fig. 3A, B), and concurrent use of these two inhibitors can effectively reverse more cell death induced by SETD8 knockdown (Fig. S2B). SETD8 knockdown induced apoptosis (Fig. 3C) and ferroptosis (Fig. 3D–F), with FTL protein significantly decreased and NOX4 protein markedly upregulated (Fig. S2C) in ES cells. Taken together, the anti-ES effect of inhibiting SETD8 with UNC0379 or genetic knockdown may be achieved by inducing apoptosis and ferroptosis.

### SETD8 exerted a pro-tumor effect in vivo

**Fig. 4:**
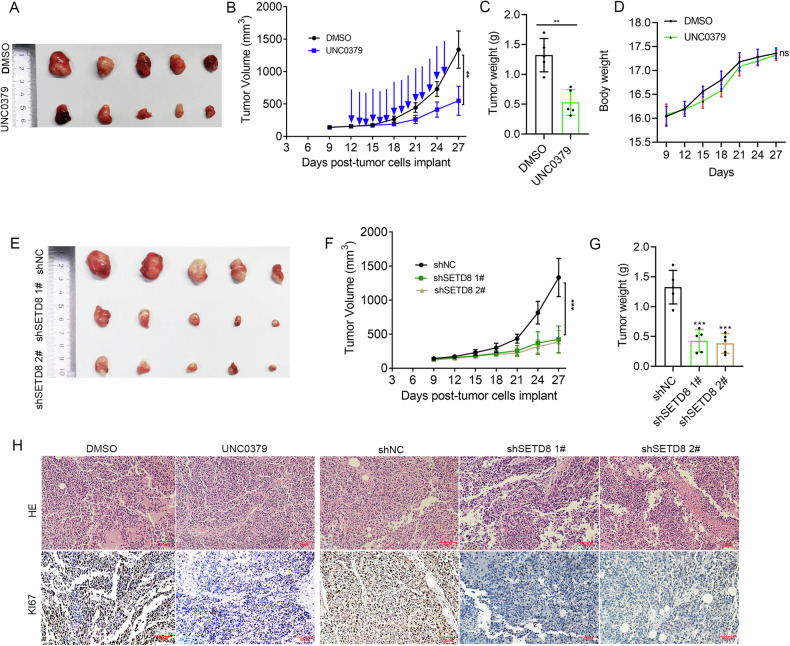
SETD8 inhibition significantly impaired ES growth in vivo. **A** Images captured on day 14 posttreatment showcase the excised tumors. **B** The xenograft tumor growth is depicted through growth curves, with blue arrows marking the days of UNC0379 administration. **C** A comparative histogram illustrates the distribution of xenograft tumor weights between the control and UNC0379 treated groups. **D** The weight changes of the mice are tracked over the course of the experiment. **E** Photographs present the tumors excised from the shNC and shSETD8 groups. **F** Growth curves of xenograft tumors from both the shNC and shSETD8 groups are displayed. **G** A histogram contrasts the weights of xenograft tumors between the shNC and shSETD8 groups. **H** HE staining and IHC images of Ki67 expression in xenograft samples from the DMSO, UNC0379, shNC and shSETD8 groups are provided. ‘ns’ denotes no statistical significance. *p < 0.05, **p < 0.01, ***p < 0.001.

To further verify the pro-tumor effect of SETD8 in vivo, nude mouse xenograft models were established using A673 cells. It was found that treatment with UNC0379 (30 mg/kg/d) for 14 days significantly reduced tumor volume and weight compared with placebo-treated controls (Fig. 4A–C). Crucially, the study demonstrated that the adverse effects associated with the treatment were manageable, with only a slight reduction in body weight being recorded, indicating a favorable tolerability profile (Fig. 4D). Consistently, the tumor growth rate under SETD8 knockdown was dramatically lower than that in the shNC group (Fig. 4E–G). The results of IHC assays also showed that there were fewer Ki67-positive cells after treatment with UNC0379 or SETD8 knockdown (Fig. 4H). Overall, SETD8 exerts a pro-tumor effect in vivo.

### SETD8 activated the MAPK pathway to inhibit apoptosis and ferroptosis of ES

**Fig. 5:**
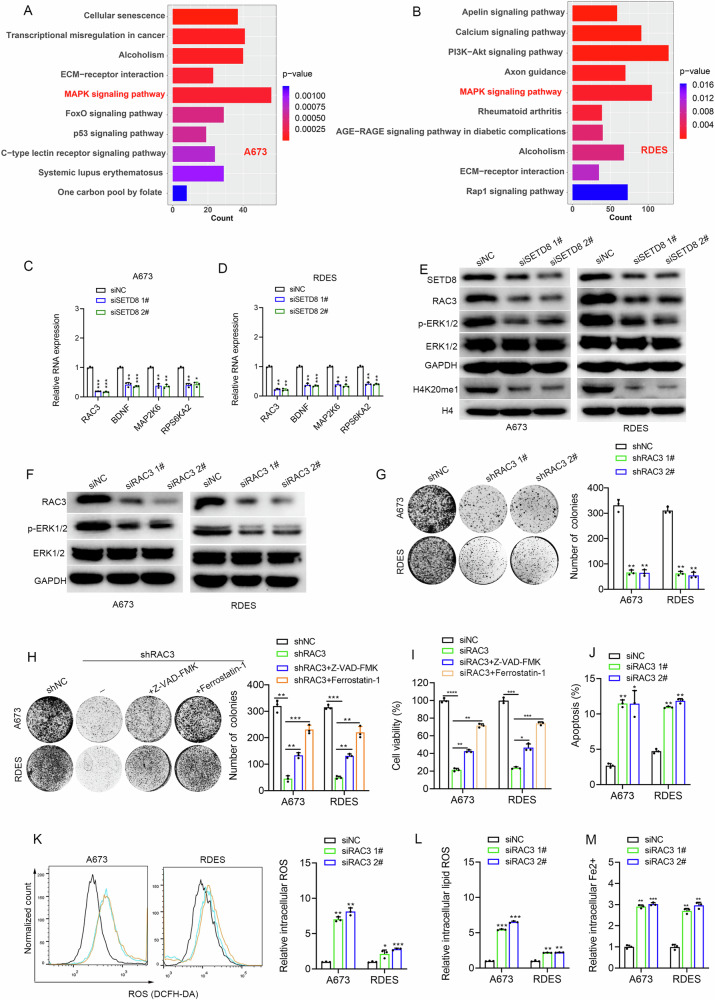
SETD8 inhibited ES cell apoptosis and ferroptosis via the MAPK pathway. **A**, **B** The KEGG pathway analysis enriched by DEGs from A673 and RDES after SETD8 suppression by siRNA. **C**, **D** mRNA expression levels of DEGs in the MAPK pathway validated in ES cells after SETD8 genetic knockdown by siRNA. **E** Results of WB analysis to confirm the protein expression changes of RAC3, ERK1/2, and p-ERK1/2 in the MAPK pathway after SETD8 inhibition. The original blots corresponding to these experiments are displayed in Fig. S10B. **F** Results of WB analysis to confirm the protein expression changes of ERK1/2 and p-ERK1/2 after RAC3 knockdown. The original blots corresponding to these experiments are displayed in Fig. S10C. **G** Colony formation assay after RAC3 knockdown in ES cells. **H** Colony formation assay determined the reproductive ability of ES cells with RAC3 knockdown in the absence or presence of indicated cell death inhibitors (10 µM Z-VAD-FMK and 10 µM Ferrostatin-1). **I** A673 and RDES cells with RAC3 knockdown in the absence or presence of indicated cell death inhibitors (10 µM Z-VAD-FMK and 10 µM Ferrostatin-1) for 48 h, cell viability was assayed with CCK-8. **J** Changes of apoptosis rates after RAC3 inhibition in ES cells. **K** Changes of the intracellular ROS levels after RAC3 inhibition in ES cells. **L** Changes of the intracellular lipid ROS levels after RAC3 inhibition in ES cells. **M** Changes of the relative intracellular Fe2+ levels in ES cells after RAC3 inhibition. *p < 0.05, **p < 0.01, ***p < 0.001, ****p < 0.0001.

RNA sequencing (RNA-seq) analysis was conducted to elucidate the regulatory mechanisms of SETD8 in ES. Compared with the siNC group, the down-regulated differentially expressed genes (DEGs) of SETD8 knockdown were enriched in the MAPK pathway (Fig. 5A, B). RT-qPCR analyses confirmed that among the genes within the MAPK pathway, *RAC3* exhibited the most pronounced downregulation in response to SETD8 knockdown (Fig. 5C, D). The data suggest that SETD8 may play a role in the activation of the MAPK pathway, thereby exerting an inhibitory effect on both apoptosis and ferroptosis in ES cells, potentially through the modulation of *RAC3* expression. The results of WB confirmed the downregulating of H4K20me1, RAC3, and p-ERK after SETD8 knockdown (Fig. 5E). As expected, inhibition of RAC3 could decrease the expression level of p-ERK (Fig. 5F), inducing apoptosis and ferroptosis (Fig. 5G–M and Fig. S3) of ES.

### SETD8 interacted with YBX1 to inhibit apoptosis and ferroptosis of ES

As an essential methyltransferase, SETD8 plays a pivotal role in catalyzing the mono-methylation of histone H4 at lysine 20, specifically when it is H4K20me1, as well as mediating the methylation of nonhistone proteins [18]. SETD8 primarily exerts its regulatory effects on target genes by mediating H4K20me1-dependent epigenetic modifications at the transcriptional level [13]. We hypothesized that SETD8 may transcriptionally enhance the expression of *RAC3* through the modification of H4K20me1. Despite the WB results demonstrated a significant reduction in H4K20me1 levels following SETD8 inhibition, ChIP-PCR results revealed that knocking down SETD8 does not affect the enrichment of H4K20me1 at the *RAC3* promoter (data not shown). These findings suggest that the regulation of *RAC3* by SETD8 is not mediated through H4K20me1-dependent epigenetic modifications.

**Fig. 6:**
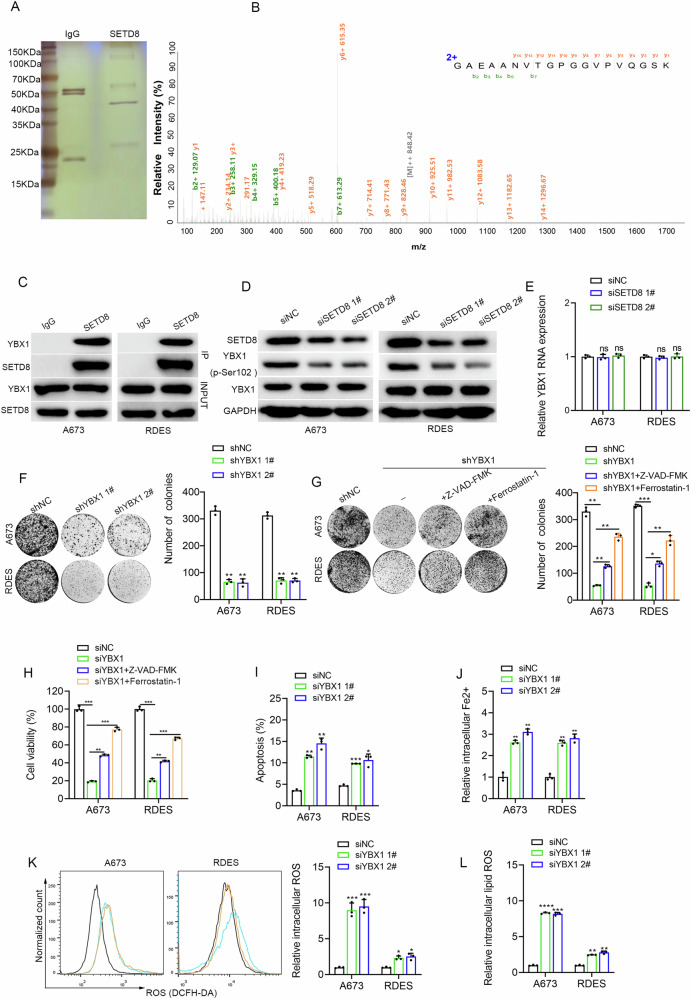
SETD8 interacted with YBX1 to inhibit apoptosis and ferroptosis in ES. **A** Silver staining demonstrated co-immunoprecipitation using an antibody against SETD8 in A673 cells. **B** Mass spectrometry data confirmed the physical association between SETD8 and YBX1 proteins. **C** Immunoprecipitation results validated the binding interaction between SETD8 and YBX1 in ES cells. The original blots corresponding to these experiments are displayed in Fig. S11A. **D** WB analysis detected changes in the expression levels of phosphorylated YBX1 at serine102 (p-Ser102 YBX1) and total YBX1 in ES cell lines following SETD8 knockdown. The original blots corresponding to these experiments are displayed in Fig. S11B. **E** Quantitative reverse transcription polymerase chain reaction (qRT-PCR) analysis revealed alterations in *YBX1* mRNA levels in ES cell lines post-SETD8 knockdown. **F** The clonogenic capacity of ES cells post YBX1 knockdown was illustrated through a colony formation assay. **G** The reproductive potential of YBX1 knockdown in ES cells was evaluated in the context of specific cell death inhibitors (10 μM Z-VAD-FMK and Ferrostatin-1) using a colony formation assay. **H** A column chart, based on CCK-8 assay results, depicted cell viability following YBX1 knockdown with or without the specified cell death inhibitors for 48 h. **I** A column chart showed the shifts in apoptosis rates across ES cell lines after YBX1 was suppressed using siRNA. **J** Relative changes in intracellular Fe2+ levels were observed in ES cells after YBX1 inhibition. **K** Intracellular ROS levels were measured in ES cells following YBX1 inhibition. **L** Intracellular lipid ROS levels were assessed in ES cells after YBX1 inhibition. Data are presented as mean ± standard deviation from three separate experiments. ‘ns’ signifies no statistical significance. *p < 0.05, **p < 0.01, ***p < 0.001.

Apart from the H4K20me1 modification, SETD8 may alternatively modulate downstream target genes through post-transcriptional regulatory mechanisms [13]. To further explore the exact regulatory mechanism of SETD8 in ES, mass spectrometry was conducted consequently. Silver staining revealed distinct bands in the 40–50 kDa range in A673 cells, which were specifically captured by the anti-SETD8 antibody (Fig. 6A) and were then cut for mass spectrometry. The results revealed that YBX1 was abundant in discrete bands (Fig. 6B). Given the known it can active MAPK pathway in a varies of cancers [19, 20], and also acted as an oncogene in ES [21], we suspected that YBX1 might be a crucial target gene of SETD8 in ES. In accordance with the findings, Co-IP assays confirmed the presence of a physical interaction between SETD8 and YBX1 (Fig. 6C).

**Fig. 7:**
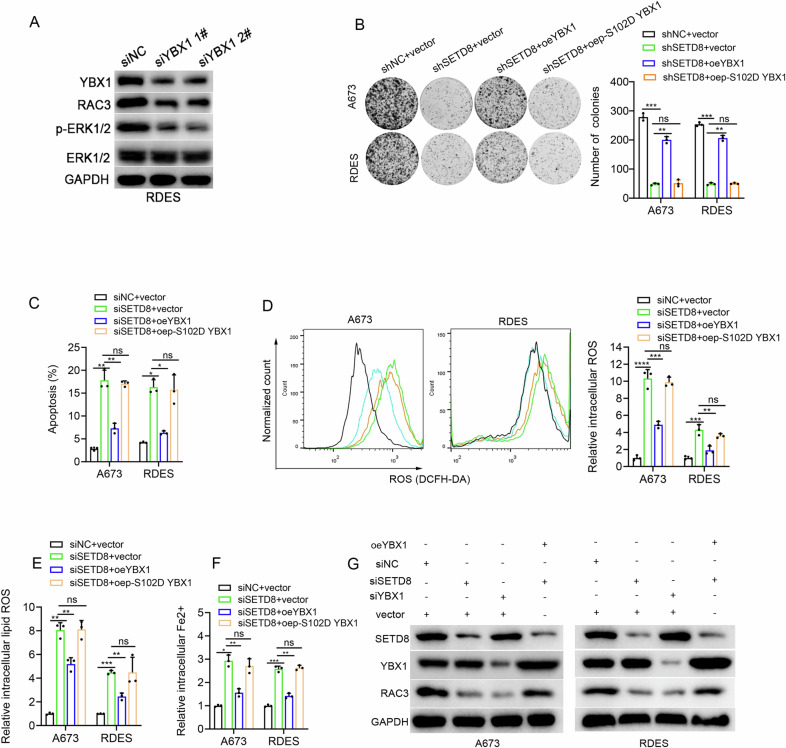
SETD8 inhibited apoptosis and ferroptosis in ES through YBX1/RAC3 axis. **A** WB analysis confirmed alterations in RAC3 protein levels following YBX1 suppression. The original blots corresponding to these experiments are displayed in Fig. S12A. **B** The colony formation assay evaluated the clonogenic capacity of ES cells with SETD8 knockdown, either alone or in combination with overexpression of YBX1 (oeYBX1) or a phosphorylation-mimicking mutant (oep-S102D YBX1). **C** A column chart illustrated variations in apoptosis rates among ES cell lines after SETD8 genetic knockdown, with or without overexpression of YBX1 or p-S102D YBX1. **D** Intracellular ROS levels were measured in ES cells after SETD8 knockdown, alone or in conjunction with overexpression of YBX1 or p-S102D YBX1. **E** Intracellular lipid ROS levels were assessed under ES cells after SETD8 knockdown, alone or in conjunction with overexpression of YBX1 or p-S102D YBX1. **F** Intracellular Fe2+ levels were examined following SETD8 knockdown, with or without overexpression of YBX1 or p-S102D YBX1. **G** WB analysis was conducted to assess RAC3 protein expression following SETD8 knockdown, either alone or with overexpression of YBX1. The original blots corresponding to these experiments are displayed in Fig. S12B. The data are expressed as the mean ± standard deviation from three independent experiments. ‘ns’ denotes no statistical significance. *p < 0.05, **p < 0.01, ***p < 0.001, ****p < 0.0001.

WB analysis indicated that the knockdown of SETD8 markedly reduced the levels of phosphorylated YBX1 at serine 102 (p-Ser102 YBX1, the activated state of YBX1) [22], but had no obvious impact on the level of total YBX1 protein (Fig. 6D). Additionally, the knockdown of SETD8 did not appear to induce obvious change in the mRNA levels of *YBX1* (Fig. 6E), which confirmed the post-transcriptional regulation of SETD8 on YBX1. Moreover, the knockdown of YBX1 significantly impaired growth (Fig. 6F) and induced apoptosis and ferroptosis (Fig. 6G–L and Fig. S4) of ES cells expectingly. According to reported literature, YBX1 is involved in regulating the MAPK pathway [20]. Our findings further substantiated that the downregulation of YBX1 led to a notable reduction in both the protein levels of RAC3 and p-ERK1/2 (Fig. 7A), which prompted us to propose that SETD8 exerts its inhibitory effects on apoptosis and ferroptosis via the YBX1/RAC3 signaling axis. Consistent with our hypothesis, the results revealed that following SETD8 knockdown, the compromised proliferative capacity and the induction of apoptosis and ferroptosis in ES cells were partially attenuated upon the re-introduction of YBX1, but S102 mutants of YBX1 abolished the various rescue effects of wild type (wt) YBX1 (Fig. 7B–F). Consistently, the downregulation of RAC3 induced by SETD8 knockdown was also partially reversed by the re-introduction of YBX1 (Fig. 7G). Collectively, these findings provide strong evidence that SETD8 modulates the proliferation, apoptosis, and ferroptosis of ES cells via the YBX1/RAC3 signaling axis.

### SETD8 promoted *RAC3* transcription through enhancing YBX1 nuclear translocation

**Fig. 8:**
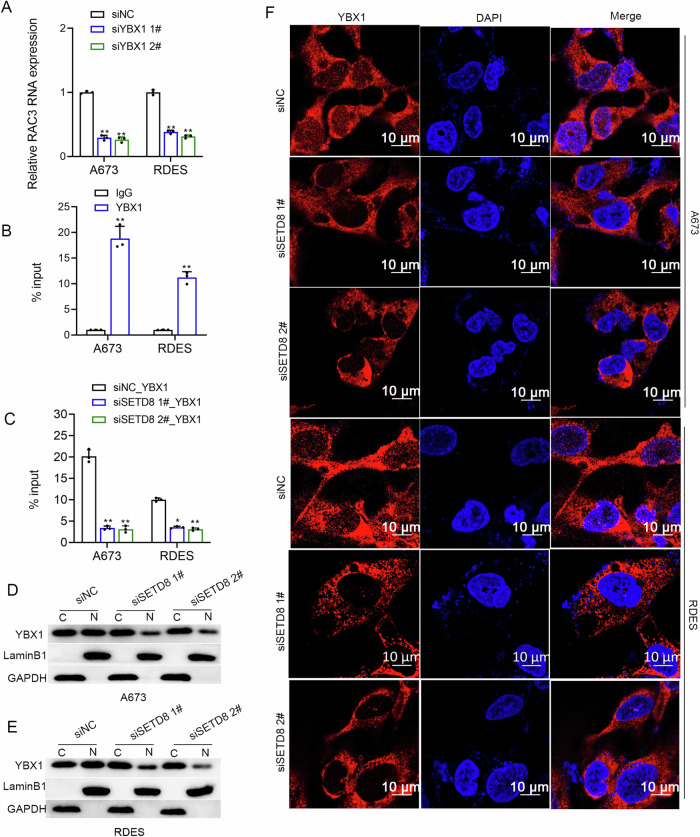
SETD8 promoted *RAC3* transcription through promoting YBX1 nuclear translocation. **A** qRT-PCR analysis verified the expression of *RAC3* mRNA subsequent to the inhibition of YBX1. **B** ChIP-PCR confirmed the presence of YBX1 bound to the *RAC3* promoter region in ES cells. **C** ChIP-qPCR was utilized to assess the alterations in YBX1 binding at the *RAC3* promoter in A673 and RDES cells with or without SETD8 knockdown. **D**, **E** WB analysis determined the distribution of YBX1 in the cytoplasmic and nuclear fractions after SETD8 knockdown for a duration of 48 h. LaminB1 served as a nuclear fraction loading control, while GAPDH was used as a cytoplasmic fraction loading control. The original blots corresponding to these experiments are displayed in Fig.S12C. **F** Immunofluorescence (IF) assays revealed the cellular localization of the YBX1 protein in ES cells following SETD8 knockdown for 48 h. Data are presented as mean ± standard deviation from three separate experiments. *p < 0.05, **p < 0.01.

As a multifunctional protein, YBX1 can transcriptionally regulate its target genes [22]. Our previous results revealed the post-transcriptional regulation of SETD8 on YBX1, and RNA-seq showed that *RAC3* was the most significantly down-regulated gene after SETD8 knockdown, but the exact molecular regulatory mechanism remains unclear. Here it was speculated that *RAC3* might be transcriptionally activated by YBX1. As expected, the results of qRT-PCR showed that YBX1 knockdown significantly decreased the expression of *RAC3* mRNA (Fig. 8A). Moreover, the results of ChIP-PCR confirmed that YBX1 was enriched in the *RAC3* promoter (Fig. 8B), while SETD8 knockdown significantly decreased the enrichment of YBX1 at the promoter of *RAC3* (Fig. 8C), which confirmed the transcriptional regulatory influence of YBX1 on the expression of *RAC3*. To further verify whether p-Ser102 YBX1 is essential for promoting the transcription of *RAC3*, we mutated p-Ser102 YBX1 and performed ChIP-PCR analysis (Fig. S5). The results indicate that p-Ser102 YBX1 is a key modification for YBX1 to promote the transcription of *RAC3*. As mentioned above, SETD8 knockdown decreased the expression of the p-Ser102 YBX1. However, how the p-Ser102 of YBX1 occurred in ES and whether this was associated with the interaction of STED8 remains unclear. According to reported literature, YBX1 is often phosphorylated by kinases, including AKT, p70S6K, and p90RSK, and then translocates into the nucleus to promote the transcription of its target genes [23]. In our present study, to reveal whether the p-Ser102 YBX1 was influenced by the interaction between STED8 and YBX1, we performed the immunoprecipitation experiments using antibodies mentioned above, and found that YBX1 can bind to AKT and SETD8, rather than p70S6K or p90RSK (Fig. S6A), and AKT inhibitors significantly reduce the phosphorylation level of p-S102 YBX1 (Fig. S6B). Given that SETD8 functions as a methyltransferase, it exerts its effects on downstream genes through methylation rather than phosphorylation [12, 13]. Therefore, we hypothesized that after SETD8 binds to YBX1, it may facilitate the binding of AKT with YBX1, which subsequently leads to the phosphorylation of YBX1 by AKT, rather than SETD8 directly mediating the phosphorylation of YBX1. Consistent with our expectation, after mutating p-Ser102, we found that it did not affect the binding between SETD8 and YBX1 (Fig. S6C). This further confirms our hypothesis that upon binding with YBX1, SETD8 promotes the phosphorylation of YBX1 by AKT, which in turn increases YBX1’s nuclear transport, thereby enabling it to perform its biological functions. Consistent with this, the binding ability of YBX1 to AKT was significantly reduced after the knockdown of SETD8 (Fig. S6D). Hence, we conclude that the binding of SETD8 with YBX1 aids in the phosphorylation of p-S102 YBX1 by AKT. We have also detected the interactions between SETD8 and YBX1 in both the cytoplasm and the nucleus (Fig. S6E, F), which may reflect the process of YBX1 dynamically translocating into the nucleus with the assistance of SETD8.

Based on the above, it was hypothesized that SETD8 enhanced YBX1 nuclear translocation, thus promoting the transcription of *RAC3*. As expected, SETD8 knockdown significantly decreased the YBX1 protein level in the nucleus (Fig. 8D–F). Altogether, these findings demonstrated that SETD8 promotes *RAC3* transcription by enhancing YBX1 nuclear translocation.

## Discussion

In this study, we observed that SETD8 was frequently upregulated in ES and correlated with clinical outcomes. Furthermore, our findings indicate that SETD8 negatively regulates apoptosis and ferroptosis in ES cells through the YBX1/RAC3 signaling axis. These results not only uncover a novel molecular mechanism by which SETD8 operates in ES but also underscore the importance of modulating apoptosis and ferroptosis as a potential therapeutic strategy in cancer treatment. Consequently, SETD8 emerges as a promising candidate for targeted therapy in ES. SETD8, also known as PR-Set7 or SET8, is a nucleosome-specific methyltransferase that catalyzes mono-methylation of H4K20me1. As previously shown, SETD8 plays important roles in a variety of essential biological processes, including cell proliferation, epithelial-mesenchymal transition, and stemness maintenance [13, 16]. Furthermore, the involvement of SETD8 in the regulation of ferroptosis resistance and apoptosis resistance has garnered intense research interest, attributable to its significant impact on tumorigenesis and tumor progression [15]. However, the role and the regulatory mechanism of SETD8 in ES remain unknown. In the current investigation, we observed that the expression of SETD8 was elevated in ES, corroborating prior findings that SETD8 is overexpressed across a spectrum of malignancies, including neuroblastoma [13], pancreatic carcinoma [12], cervical cancer [24], multiple myeloma [25], and colorectal cancer [14]. Additionally, our findings indicate a strong correlation between elevated SETD8 expression and reduced OS and EFS in patients with ES, underscoring the clinical relevance of SETD8 in this context. To our knowledge, this is the inaugural study to document the upregulation of SETD*8* in ES. Nonetheless, it is imperative to extend the sample size to robustly validate this observation.

In addition, the establishment of experimental models provided further substantiation for our investigation. Our findings revealed that the downregulation of SETD8 markedly compromised the proliferative capacity of ES cells, while simultaneously triggering apoptotic and ferroptotic processes. These outcomes are in alignment with prior studies, which have consistently implicated SETD8 as a proto-oncogene in a diverse array of malignancies, including but not limited to colorectal, neuroblastoma, pancreatic, breast, cervical cancers, and multiple myeloma [26]. For instance, in the context of colorectal cancer, SETD8 has been shown to facilitate carcinogenesis by modulating the expression levels of PCNA [14], thereby disrupting the normal cellular regulatory mechanisms. Similarly, in high-risk neuroblastoma, SETD8 augments the proliferative capacity of tumor cells and confers resistance to apoptosis through the epigenetic silencing of the tumor protein p53 [13]; and it inhibits ferroptosis in pancreatic cancer cells through the downregulation of the RRAD [15]. These findings underscore the multifaceted role of SETD8 in the pathogenesis of various cancers and its potential as a therapeutic target. In alignment with prior research, we demonstrate that the reduction of SETD8 expression substantially triggered cell growth arrest, apoptosis, and ferroptosis in ES cells. To the best of our knowledge, this investigation represents the inaugural evidence elucidating that SETD8 exerts an anti-apoptotic and ferroptosis-inhibitory effect on ES cells by facilitating the nuclear translocation of YBX1.

The literature indicates that elevated YBX1 expression is a common phenomenon across a spectrum of human malignancies. This includes, but is not limited to, pancreatic cancer [27], hepatocellular carcinoma [28], myeloid leukemia [29], breast cancer [30], prostate cancer [30], ES, osteosarcoma, and rhabdomyosarcoma [21], which contributes to tumor cell proliferation, metastasis, drug resistance, apoptosis resistance and stemness maintenance. For instance, in the context of myeloid leukemia, YBX1 has been shown to contribute to the survival of leukemia cells by selectively stabilizing Bcl-2 protein levels through an m6A-dependent regulatory mechanism, and enhances the HIF-1α protein expression by directly binding to and activating translation of *HIF-1α* messages [29]; YBX1 plays a critical role in the invasive and metastatic processes of breast cancer through its regulatory influence on MMP1 and beta-catenin [31]; and it promotes prostate cancer progression through Raf/MEK/ERK signaling [32]. In this investigation, we observed that YBX1 facilitated the activation of the MAPK pathway by transcriptionally modulating the expression of *RAC3*, enhancing apoptosis and ferroptosis resistance in ES. To further observe whether MAPK can regulate the phosphorylation of Ser102, we performed validations and found that the phosphorylation level of p-S102 YBX1 did not change significantly after inhibiting MAPK (Fig. S7). This novel finding aligns with the established literature that has consistently implicated YBX1 in the upregulation of the MAPK pathway across a range of human cancers [32, 33]. Even though Chansky et al. have reported the tumorigenic role of YBX1 in ES [21], the ferroptosis resistance and apoptosis resistance regulation of YBX1 in ES was initially found in our study. Especially, it was found for the first time that the MAPK activation of YBX1 was dependent on the interaction with SETD8, which led to the nuclear translocation of YBX1 and then activated in ES. Nonetheless, the precise molecular mechanisms by which SETD8 promotes the nuclear translocation of YBX1 are yet to be fully elucidated and require additional research for a comprehensive understanding of this process.

RAC3 is a member of the SRC/p160 coactivator family that also includes SRC-1 (NCoA1) and SRC-2 (GRIP1, TIF2, and NCoA2) [34]. RAC3 expression is frequently elevated in a diverse range of human cancers, including colon carcinoma, lung adenocarcinoma, breast cancer, pancreatic cancer, and bladder cancer, which has been verified to be a crucial regulator in tumor initiation [35]. For example, RAC3 promotes the growth of breast cancer cells through the p21-activated kinase-dependent pathway [36], enhances the proliferation of cancer cells in vitro and in vivo, and facilitates proliferation, migration, and invasion of bladder cancer cells through the PYCR1/JAK/STAT axis [37]. However, its role in ES is still unknown. Herein, we report a novel observation that RAC3 exerts anti-apoptotic and anti-ferroptotic effects in ES cells. This discovery aligns with the established notion that RAC3 functions as an oncogene across a spectrum of human cancers [36, 38]. Consistent with the mode of cell death induced by SETD8 knockdown, the combined use of ferroptosis inhibitors and apoptosis inhibitors can more effectively reverse cell death caused by the knockdown of YBX1 and RAC3 (Fig. S8). However, these two types of inhibitors cannot completely reverse the cell death induced by the intervention of the SETD8/YBX1/RAC3 axis, which suggests that interfering with the SETD8/YBX1/RAC3 axis may also cause other modes of cell death. One thing can be certain is that the intervention of the SETD8/YBX1/RAC3 axis mainly induces apoptosis and ferroptosis in ES cells.

## Conclusions

SETD8 serves as an unfavorable indicator of prognosis for ES patients, as it stimulates the growth of cancer cells and curbs both apoptosis and ferroptosis through the YBX1/RAC3 pathway. Consequently, SETD8 could emerge as a promising target for therapeutic intervention in ES.

## Materials and methods

### Antibodies and reagents

Antibodies targeting PARP (66520-1-Ig), Ki67 (27309-1-AP), YBX1 (20339-1-AP), ERK1/2 (11257-1-AP), and phosphorylated ERK1/2 (p-ERK1/2, 28733-1-AP) were sourced from Proteintech. The GAPDH antibody (AC001) was procured from ABclonal. The antibody against SETD8 (PA5-112135) was purchased from Thermo Fisher Scientific. The antibody against phospho-Ser102-YBX1 (p-Ser102 YBX1, YP0489) was purchased from ImmunoWay. The antibody against RAC3 was purchased from HuaBio Technology. The small-molecule library, UNC0379, 3-Methyladenine, necrostatin-1, Z-VAD-FMK, deferoxamine, and Ferrostatin-1 were purchased from Topscience.

### Patients and tissues

Tissue samples were collected from individuals at Sun Yat-sen University, Sun Yat-sen Memorial Hospital who had received a pathological diagnosis of ES between May 2002 and November 2022. The criteria for participation included: (1) individuals under the age of 18, (2) those with a confirmed pathological diagnosis of ES, and (3) those with comprehensive medical documentation and subsequent follow-up information. Consent was secured from every participant, and the research protocols were executed with the authorization and scrutiny of the Ethical Review Board at Sun Yat-sen Memorial Hospital, Sun Yat-sen University.

### Immunohistochemistry (IHC) assay

IHC assay was performed according to the standard protocol. Briefly, the tissue sections were deparaffinized with xylene, and retrieved by boiling in sodium citrate buffer. Then the sections were incubated with anti-human SETD8 and Ki67 primary antibodies at 4 °C overnight. The next day, the sections were incubated with goat anti-mouse/rabbit secondary antibodies for 30 min at 37 °C, followed by staining with DAB Detection Kit (GeneTech, Shanghai, China). The quantification results of protein expression were assessed independently by two pathologists, and the mean scores were given. A score of 0–4 points and 5–9 points was considered as low expression and high expression, respectively.

### Cell culture

The RDES and SKNMC human ES cell lines, along with BMSC, were procured from Jennio Biotech Co., Ltd. Additionally, the A673 and SK-NEP-1 human ES cell lines were sourced from Procell, Wuhan, China. These cell lines were maintained in a full-strength growth medium enriched with either 10% or 15% fetal bovine serum, in accordance with the supplier’s instructions. The cells were cultivated in an environment at 37 °C with controlled humidity and a 5% CO_2_ atmosphere.

### High-throughput drug library screening

High-throughput screening for drug discovery was performed on a small-molecule library including 294 small molecular compounds (Topscience). Briefly, A673 cells were inoculated in 96-well plates at a density of 6000 cells/well. 12 h later, the cells were incubated with each drug at a working concentration of 2 μM for 72 h, with three replicates for each drug. Then the cell viability was assessed by CCK-8 assays. A cell survival rate of less than 40% indicated that the drugs were effective.

### Total ROS assay

The total intracellular ROS level was measured using the ROS Detection Kit (Beyotime, S0033S). In brief, ES cells were incubated with 10 μM DCFH-DA for 20 min at 37 °C. After excess DCFH-DA was washed away with a serum-free medium, the fluorescence intensity was detected by flow cytometry.

### Lipid ROS assay

The lipid ROS level in cells was measured using C11-BODIPY (Thermo Fisher Scientific) as a fluorescent probe according to the manufacturer’s instructions. In brief, ES cells were harvested, resuspended, and washed, followed by stained with 1 μM C11-BODIPY for 30 min. Then the fluorescence intensity was detected by a flow cytometer.

### Iron assay

The total cellular iron level was measured using Iron Assay Kit (Solarbio, BC5415) following the manufacturer’s instructions. Specifically, the cells were rapidly homogenized in Reagent I, sonicated on ice for 5 min, and centrifuged at 10,000 × *g* and 4 °C for 10 min. Then 200 μL of samples and 100 μL of Reagent II were added into a 1.5 mL EP tube, fully mixed, and incubated for 10 min at 37 °C. Then the samples were added with 100 μL of CHCL3, shaken for 5 min, and centrifuged at 12,000 × *g* for 10 min at room temperature. 200 μL of cell samples and diluted standard samples were added into 96-well plates, and then the absorbance was measured at 593 nm with a microplate reader. The corresponding iron level in each sample was calculated based on the standard curves.

### RNA isolation and reverse transcription-quantitative polymerase chain reaction (RT-qPCR)

Total RNA was extracted from cells with TRIzol reagent (TaKaRa Bio Inc., Kusatsu, Japan) and synthesized into cDNA with SuperScript II Reverse Transcriptase. Then RT-qPCR was performed using SYBR GreenER qPCR SuperMix. The cycle threshold (Ct) value of each gene was normalized to that of GAPDH, and the relative gene expression was analyzed by the 2-ΔΔCt method. PCR amplification was performed in a 10 μL qPCR mixture using the iTaq Universal SYBR Green One-step Kit (Dongsheng Biotech Co., Ltd, V5012), with three replicates for each sample. The sequences of primers for RT-qPCR are listed in Table S1.

### Cell transfection and viral infection

The small interfering RNAs (siRNAs), SETD8 siRNA (siSETD8), YBX1 siRNA (siYBX1), RAC3 siRNA (siRAC3), and synthetic sequence-scrambled siRNA (siNC) used for the transient knockdown assay were provided by GenePharma (Shanghai, China). The short hairpin RNAs (shRNAs) of SETD8, YBX1, and RAC3 were purchased from GeneCopoeia (Guangzhou, China). The transfection and infection procedures were conducted following the manufacturer’s instructions. Then stable cell lines were selected using the appropriate antibiotics for at least 48 h after transfection.

### Cell death manner assay

To investigate the inhibitory mechanism of UNC0379 in ES cell proliferation, the cell death manner was analyzed. The cell autophagy inhibitor 3-Methyladenine, cell necrosis inhibitor necrostatin-1, cell apoptosis inhibitor Z-VAD-FMK, and ferroptosis inhibitors deferoxamine and Ferrostatin-1 were purchased and stored at −20 °C. Firstly, ES cells were seeded into 96-well plates at a density of 6000 cells/well. 12 h later, different concentrations of UNC0379 were added. Then 5 mM 3-Methyladenine, 10 µM necrostatin-1, 10 µM Z-VAD-FMK, 200 µM deferoxamine, and 10 µM Ferrostatin-1 were added and co-incubated with UNC0379, respectively, for 72 h. CCK-8 solution was added and incubated at 37 °C for 3 h. Finally, the absorbance was measured at 450 nm, and the inhibitory percentage was calculated.

### Colony formation assay

ES cells were seeded into 6-well plates at a density of 6000 cells/2 mL/well. After 12 h, compounds at different concentrations were added and incubated for 10 days at 37 °C and 5% CO_2_. Finally, the cells were fixed with 4% paraformaldehyde, stained with 0.1% crystal violet, and counted.

### Apoptosis assay

In brief, 4 × 10^5^ cells were collected, washed with cold PBS, and resuspended in 400 μL of binding buffer. Then they were treated with 5 μL of Annexin V-Alexa Fluor 647 for 5 min and with 10 μL of 7-AAD for 2 min away from light, followed by flow cytometry within 1 h.

### Western blotting (WB)

A673 and RDES cells were lysed with RIPA lysis buffer containing protease inhibitor cocktail (KeyGene, Shanghai, China) for 30 min on ice to extract the protein. The protein was separated by sodium dodecyl sulfate-polyacrylamide gel electrophoresis (SDS-PAGE) and transferred onto a polyvinylidene difluoride (PVDF) membrane (Sigma–Aldrich). Then the membrane was blocked with 5% skim milk for 1 h at room temperature, and incubated with primary antibodies against SETD8, YBX1, p-Ser102 YBX1, RAC3, ERK1/2, and p-ERK1/2 at 4 °C overnight on a rotary shaker. After washing three times with TBST, the membrane was incubated with rabbit anti-mouse secondary antibodies (1:10,000) for 1 h at room temperature. Finally, the membrane was washed for three times and developed with the ECL reagent.

### Co-immunoprecipitation (Co-IP) assay

The cells were lysed with cell lysis buffer containing protease inhibitor cocktail. The lysates were incubated with the corresponding primary antibodies overnight, added with an equal number of beads (Thermo Fisher Scientific), and incubated for 4 h. Then the beads were washed and the immunoprecipitated samples were denatured with SDS-loading buffer, followed by WB. Whole-cell lysates served as an input control, and normal IgG as a negative control.

### Chromatin immunoprecipitation (ChIP) assay

ChIP assay was performed with ChIP Kit (Cell Signal Technology, Cat. No. #9005, Danvers, MA, USA) according to the manufacturer’s instructions. The percentage of copies of *RAC3* gene promoter binding to YBX1 was quantified by qPCR using *RAC3* promoter-specific primers.

### In vivo xenograft mouse models

The study protocol was approved by the Committee on the Use of Live Animals in Teaching and Research of Sun Yat-sen University and implemented in accordance with the guidelines. Specifically, 3–4-week-old female nude mice were injected subcutaneously with 8 × 10^6^ A673 cells into the right flank. Tumor volumes were calculated using the formula for the volume of ellipsoid (1/2 × D1 × D2 × D2), where D1 and D2 were the maximum diameter and the minimum diameter of tumors, respectively. The growth and volume of the tumor were monitored using a caliper every 3 days for up to 4 weeks.

### Statistical analysis

Data were analyzed employing GraphPad Prism version 8 or SPSS version 20 software packages. Continuous data were analyzed by computing the mean and standard deviation, with variances being tested using either the Student’s *t*-test for two-group comparisons or the one-way ANOVA for more than two groups. Categorical variable differences were evaluated using the chi-square test. Survival statistics were determined utilizing the Kaplan–Meier estimator and differences were examined with the log-rank test. Statistical tests were conducted bilaterally, with a *p*-value threshold of 0.05 to denote statistical significance.

## Supplementary information

supplementary material

The sequence of primers used in this study

## Data Availability

All data generated or analyzed during this study are included in this published article.
