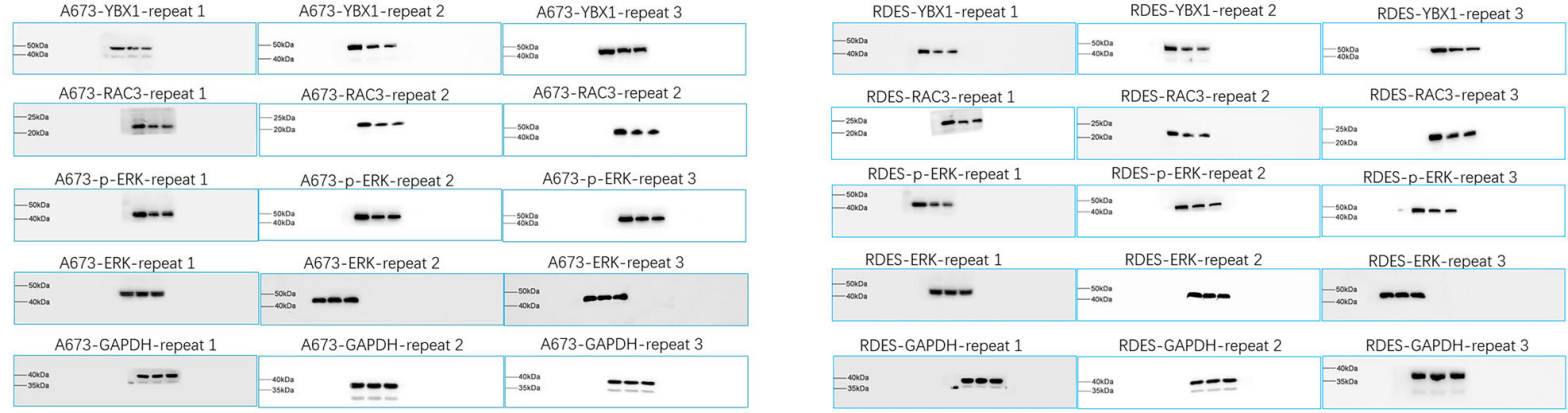# Correction: SETD8 inhibits apoptosis and ferroptosis of Ewing’s sarcoma through YBX1/RAC3 axis

**DOI:** 10.1038/s41419-025-07483-6

**Published:** 2025-04-28

**Authors:** Huimou Chen, Jing Hu, Xilin Xiong, Hongling Chen, Qiaofang Liao, Biaojun Lin, Yusong Chen, Yanting Peng, Yang Li, Di Cheng, Zhihua Li

**Affiliations:** 1 Department of Oncology, Sun Yat-sen Memorial Hospital of Sun Yat-sen University, No. 107 Yanjiang Road, Guangzhou, 510120 China; 2 Department of Clinical Laboratory, The Sixth Affiliated Hospital of Sun Yat-sen University, Guangzhou, China; 3 Biomedical Innovation Center, The Sixth Affiliated Hospital of Sun Yat-sen University, Guangzhou, China; 4 Department of Oncology, Medical Centre of Pediatric, Sun Yat-sen Memorial Hospital, Sun Yat-sen University, No. 107 Yanjiang Road, Guangzhou, 510120 China; 5 Department of Clinical Laboratory, Maoming People’s Hospital, Maoming, Guangdong 525000 China; 6 Department of Oncology, Huizhou First Hospital, Huizhou, Guangdong 516000 China

**Keywords:** Paediatric cancer, Targeted therapies

Correction to: *Cell Death & Disease* 10.1038/s41419-024-06882-5, published online 10 July 2024

The authors sincerely regret the errors in Figure 2E and Figure 7A of the published article. Specifically, incorrect images were inadvertently reused during the figure assembly process.

In Figure 2E, the colony formation image of A673 was placed by mistake due to our oversight in organizing images while dealing with a large number of pictures, leading to the incorrect copying of images. Similarly, in Figure 7A, an error occurred during the combination of western blot bands, resulting in the incorrect copying of the A673-H4K20me1 band in Figure 5E to the RDES-YBX1 band in Figure 7A.

correct Figure 2E
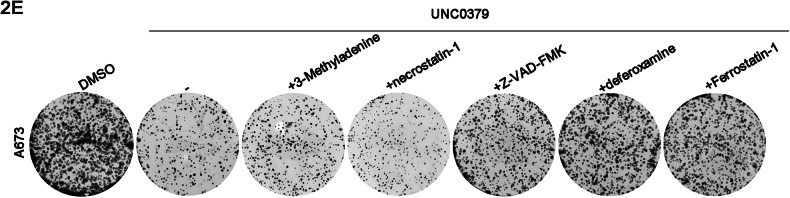


Original Figure 2E
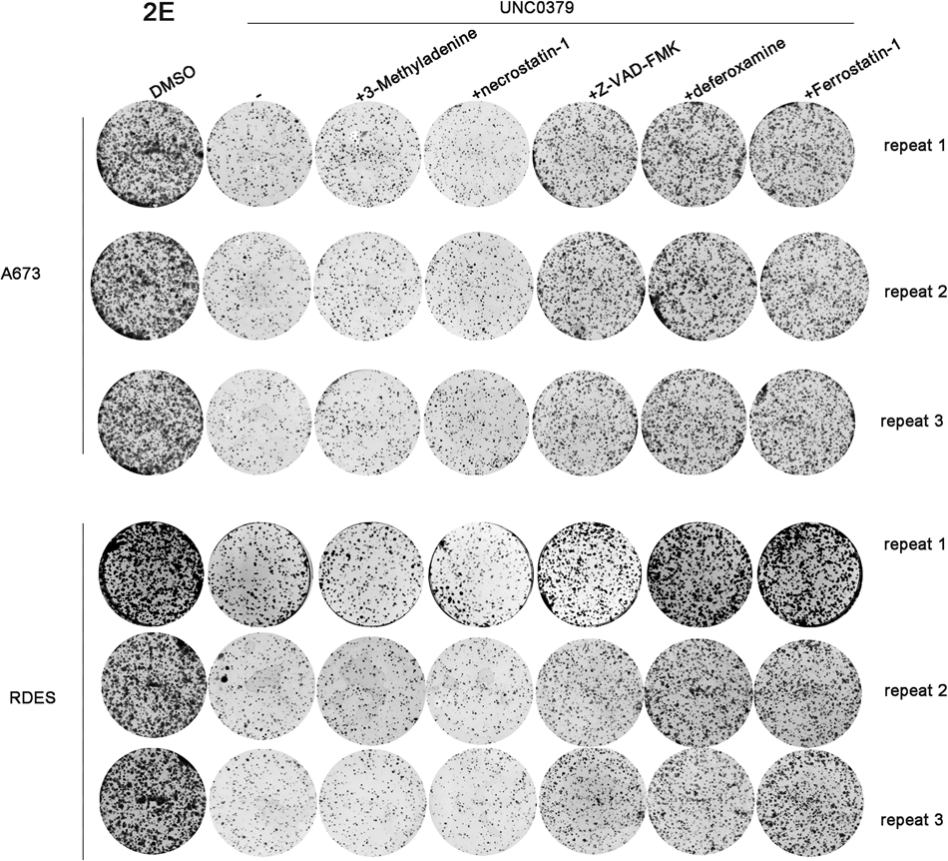


Correct Figure 7A
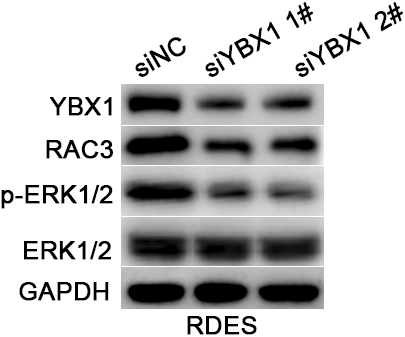


Original Figure 7A