# Supplementary material for: SETD8 inhibits apoptosis and ferroptosis of Ewing’s sarcoma through YBX1/RAC3 axis
Source: Cell Death Dis. 2024 Jul 10;15(7):494. doi: 10.1038/s41419-024-06882-5 (PMC11237091; doi:10.1038/s41419-024-06882-5)

**Fig S1. A drug screening process utilizing a library of 294 small molecule compounds has identified 31 compounds exhibiting anti-Ewing's sarcoma activity.**

**
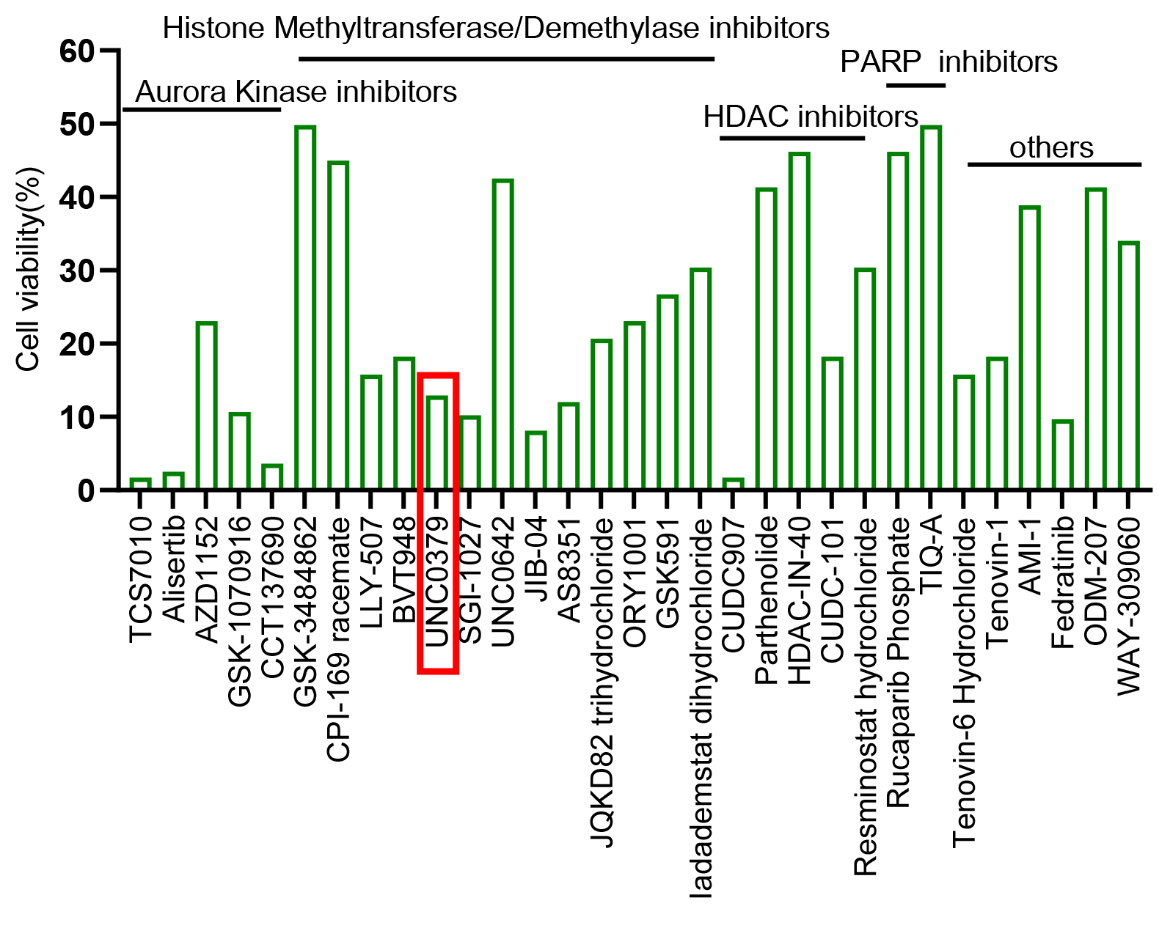
**

**Fig S2. UNC0379 or the knockdown of SETD8 significantly induces apoptosis and ferroptosis in Ewing's sarcoma.**

**
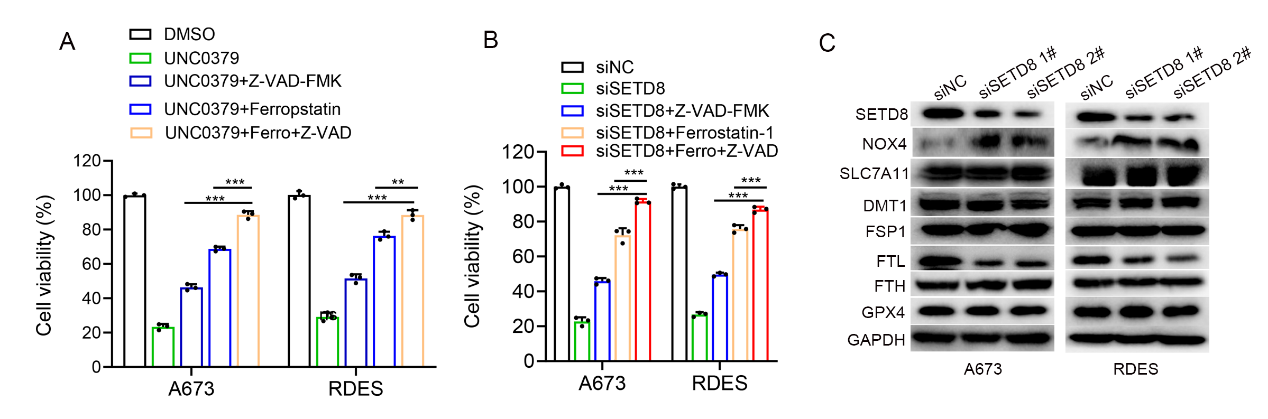
**

(A) The use of apoptosis inhibitors in conjunction with ferroptosis inhibitors can more effectively ameliorate the reduction in ES cell activity induced by UNC0379. (B) The use of apoptosis inhibitors in conjunction with ferroptosis inhibitors can more effectively ameliorate the reduction in ES cell activity induced by SETD8 knockdown. (C) Knockdown of SETD8 leads to a significant downregulation of FTL and upregulation of NOX4 protein expression. The original Western blot images are displayed in Fig. S13. ***p*<0.01, ****p*<0.001.

**Fig S3. Knockdown of RAC3 leads to a significant downregulation of FTL and upregulation of NOX4 protein expression.**

**
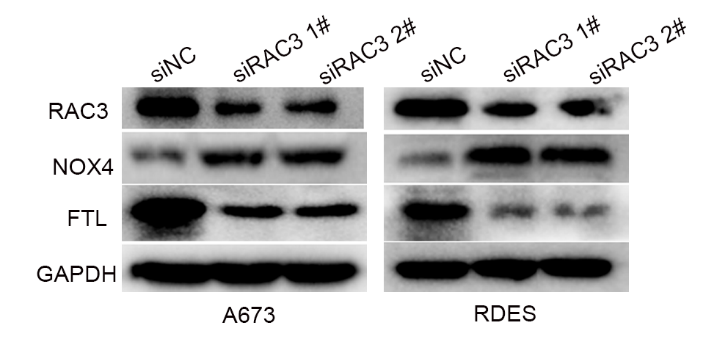
**

The corresponding original western blots were showed in Fig. S14.

**Fig S4. Knockdown of YBX1 leads to a significant downregulation of FTL and upregulation of NOX4 protein expression.**

**
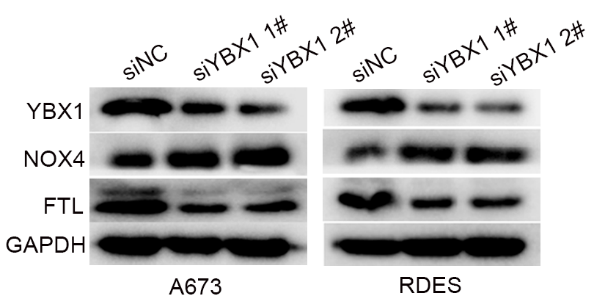
**

The corresponding original western blots were showed in Fig. S15.

**Fig S5. YBX1 promotes *RAC3* transcription in the form of p-S102 phosphorylation.**

**
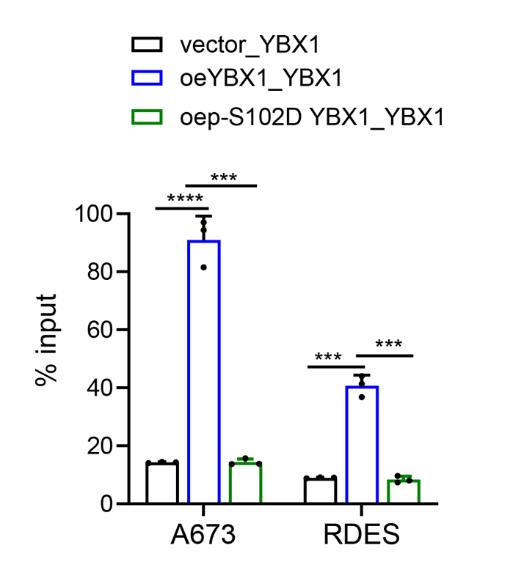
**

****p*<0.001, *****p*<0.0001.

**Fig S6. The binding of SETD8 with YBX1 aids in the phosphorylation of p-S102 YBX1 by AKT.**

**
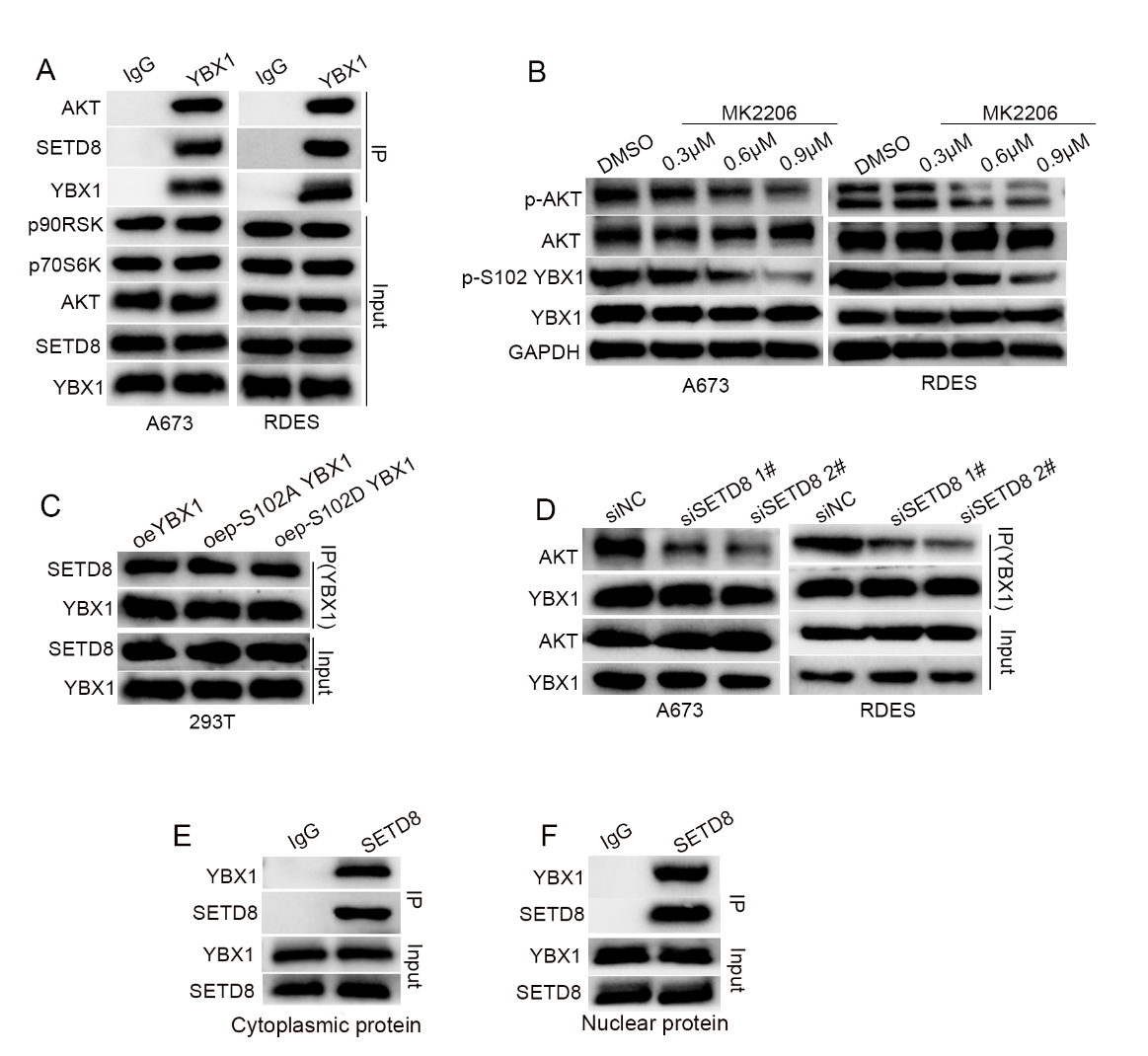
**

(A)The CoIP experiment confirmed that YBX1 forms a complex with both SETD8 and AKT. The corresponding original western blots were showed in Fig. S16A.

(B) AKT inhibitors MK2206 significantly reduce the phosphorylation level of p-S102 YBX1. The corresponding original western blots were showed in Fig. S16B.

(C) The mutation of p-S102 YBX1 did not affect the binding between SETD8 and YBX1. The corresponding original western blots were showed in Fig. S16C.

(D) The binding ability of YBX1 to AKT was significantly reduced after the knockdown of SETD8. The corresponding original western blots were showed in Fig. S16D. (E, F) The interaction between YBX1 and SETD8 can occur both in the cytoplasm and in the nucleus was indentified in A673. The corresponding original western blots were showed in Fig. S16E, F.

**Fig S7. MAPK kinases have no effect on p-S102 phosphorylated YBX1.**

**

**

The corresponding original western blots were showed in Fig. S17.

**Fig S8. The use of apoptosis inhibitors in conjunction with ferroptosis inhibitors can more effectively ameliorate the reduction in ES cell activity induced by YBX1 or RAC3 knockdown.**

**
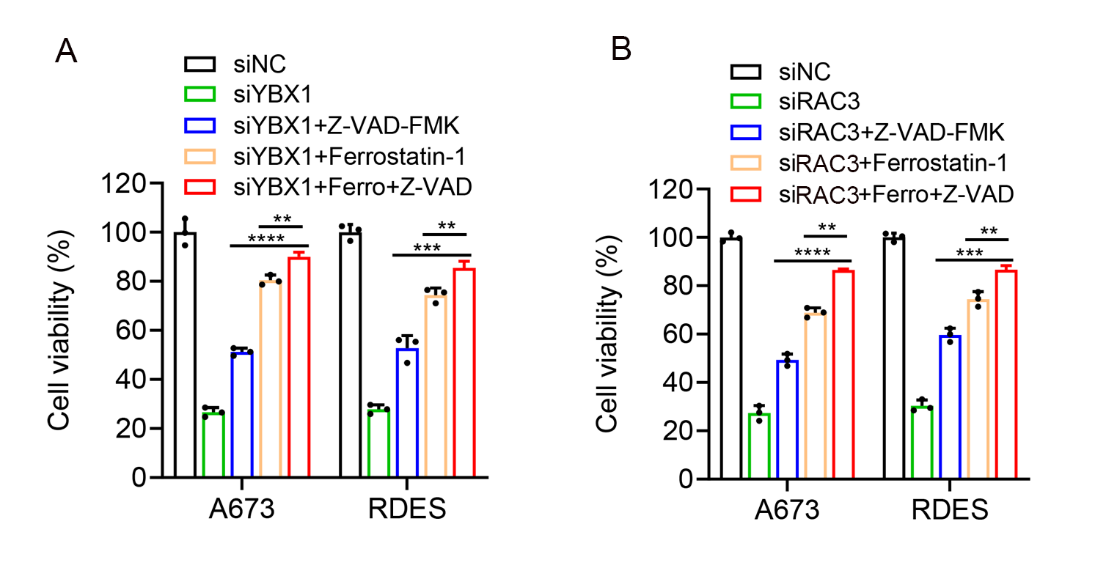
**

(A) The use of apoptosis inhibitors in conjunction with ferroptosis inhibitors can more effectively ameliorate the reduction in Ewing's sarcoma cell activity induced by YBX1 knockdown. (B) The use of apoptosis inhibitors in conjunction with ferroptosis inhibitors can more effectively ameliorate the reduction in Ewing's sarcoma cell activity induced by RAC3 knockdown. ***p*<0.01,****p*<0.001,*****p*<0.0001.

**Fig S9. Efficiency of siRNA, shRNA and plasmid.**

**
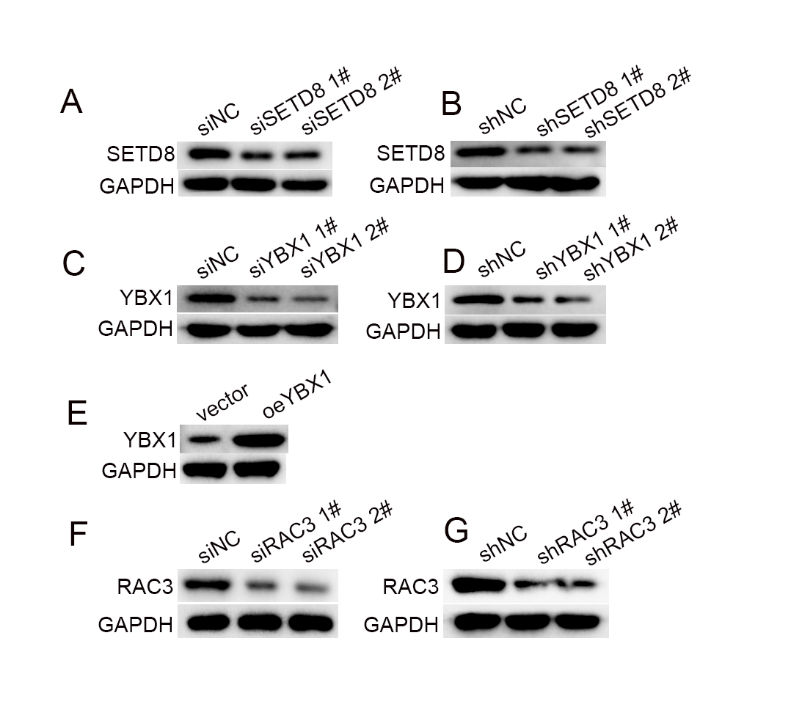
**

(A) WB analysis of the efficiency of siSETD8 in A673. (B) WB analysis of the efficiency of shSETD8 in A673. (C) WB analysis of the efficiency of siYBX1 in A673. (D) WB analysis of the efficiency of shYBX1 in A673. (E) WB analysis of the efficiency of the YBX1 overexpressed plasmid in A673. (F) WB analysis of the efficiency of siRAC3 in A673. (G) WB analysis of the efficiency of shRAC3 in A673. The corresponding original western blots were showed in Fig. S18.

**Fig S10. Original full length western blots for Fig 2A, Fig 5E and Fig 5F .**

**
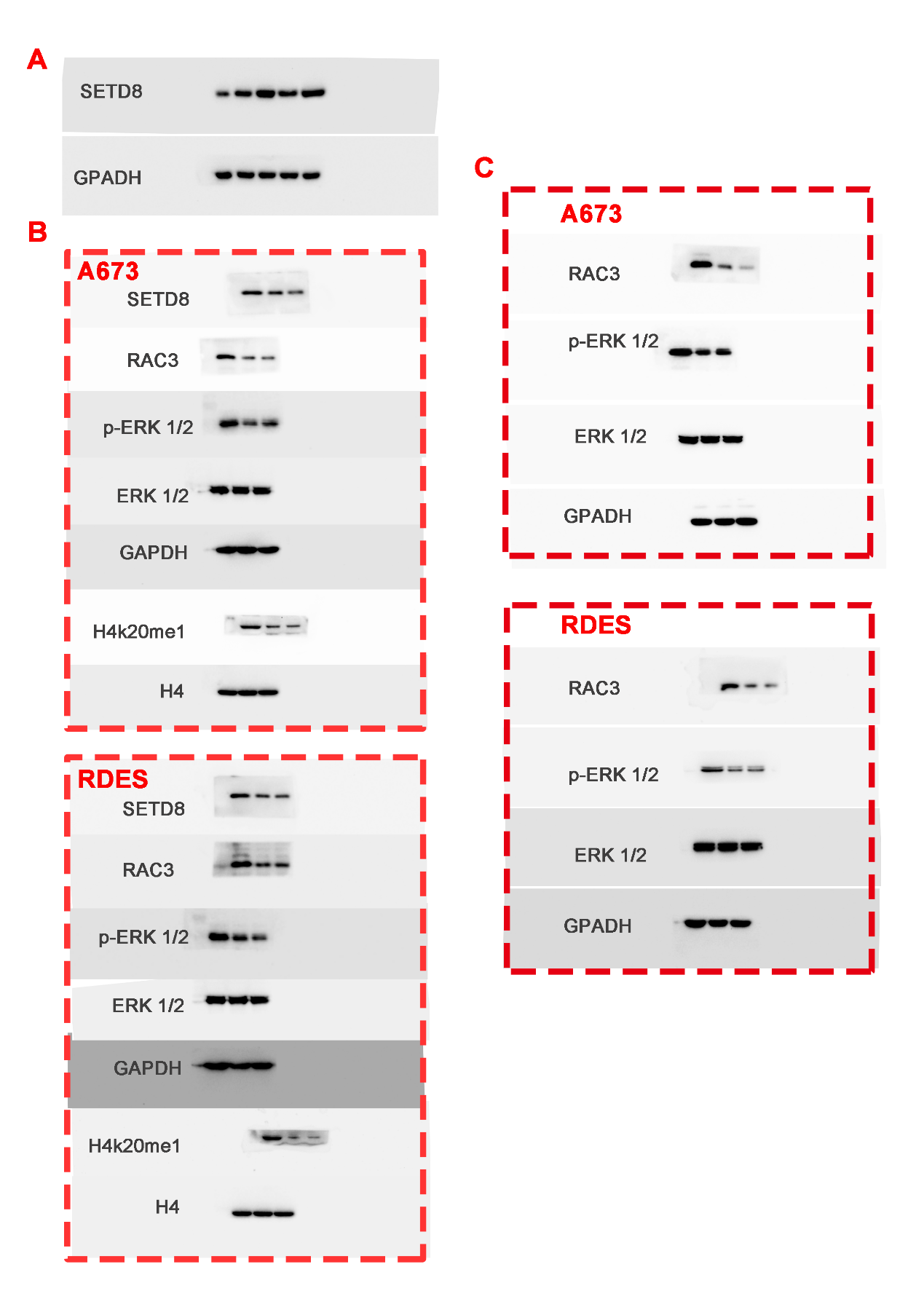
**

**Fig S11. Original full length western blots for Fig 6C and Fig 6D.**

**
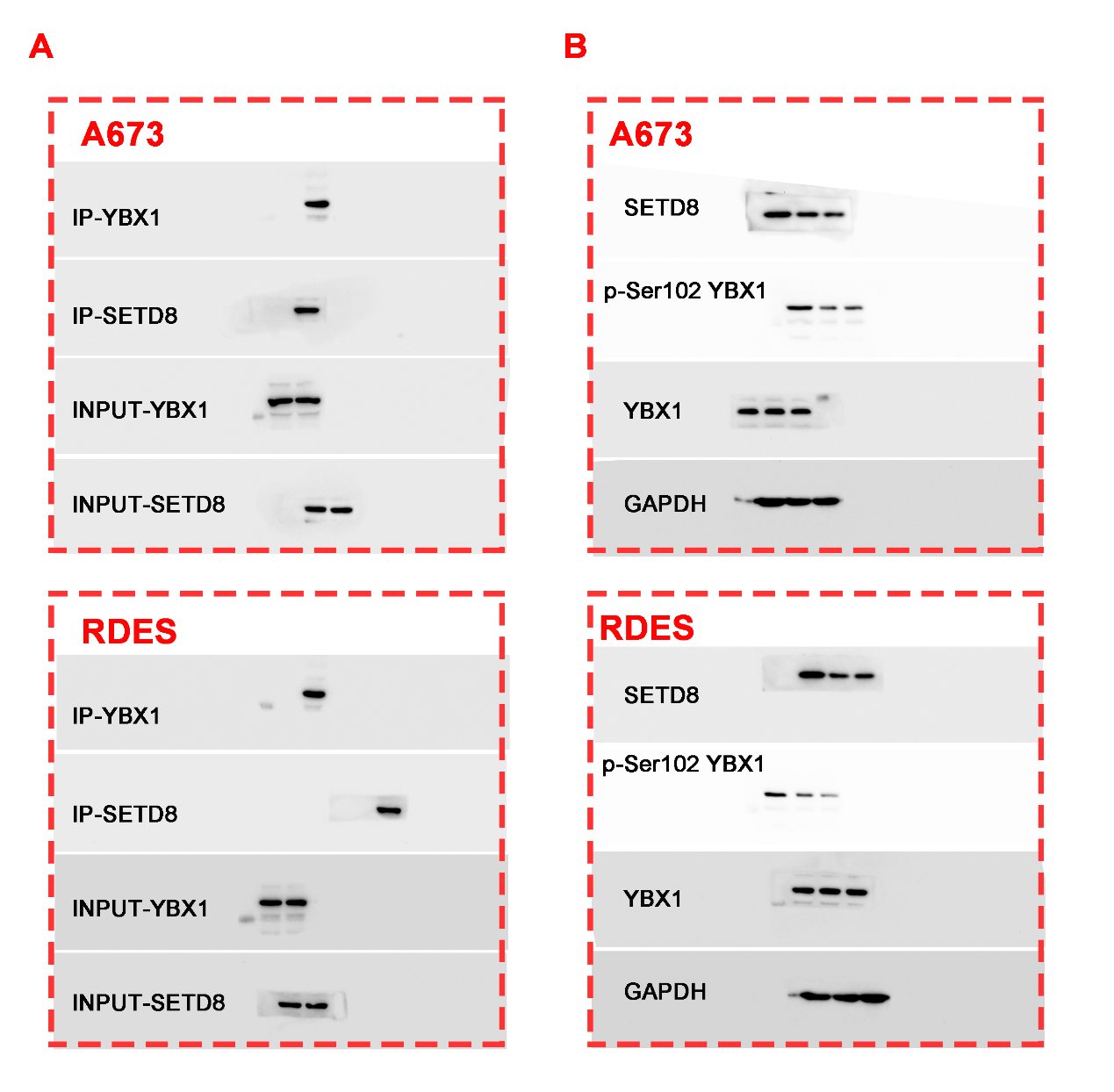
**

**Fig S12. Original full length western blots for Fig7A, Fig 7G, Fig 8D and Fig 8E.**

**
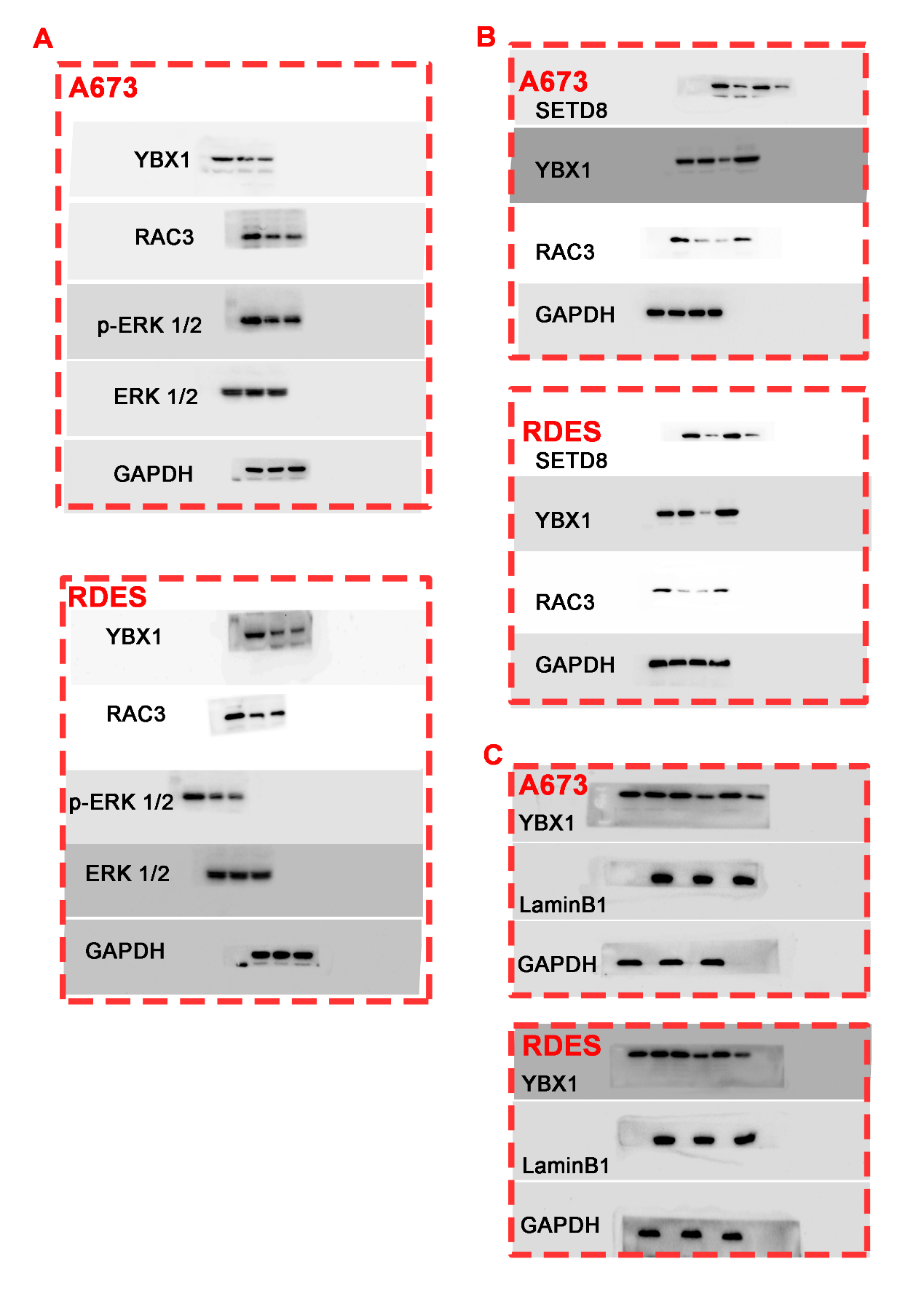
**

**Fig S13. Original full length western blots for Fig S2.**

**
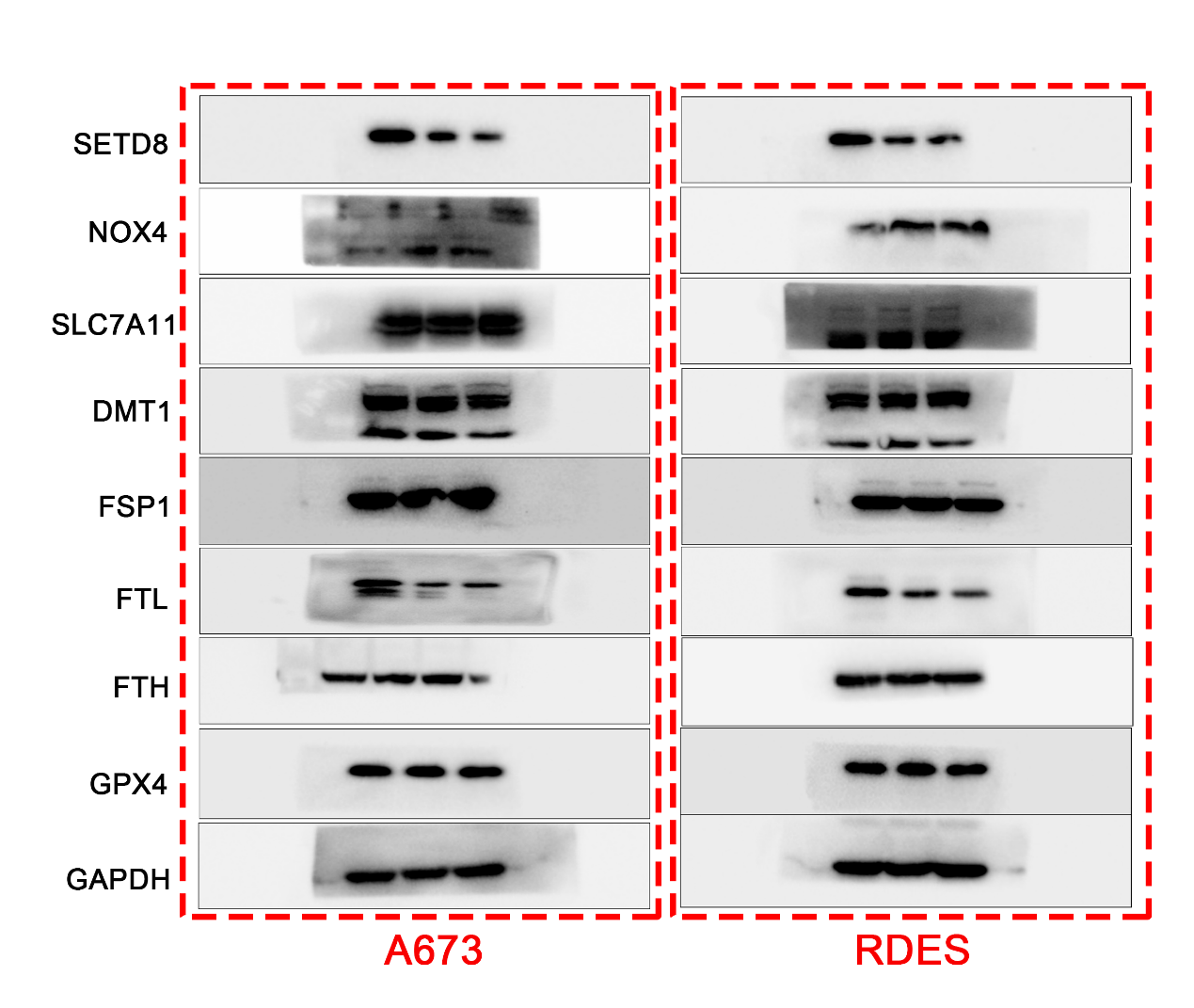
**

**Fig S14. Original full length western blots for Fig S3.**

**
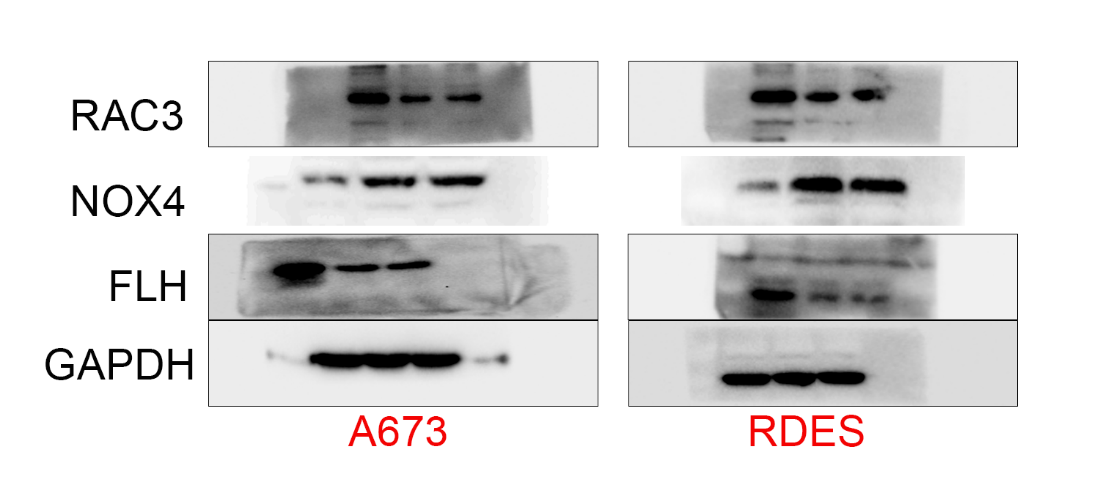
**

**Fig S15. Original full length western blots for Fig S4.**

**
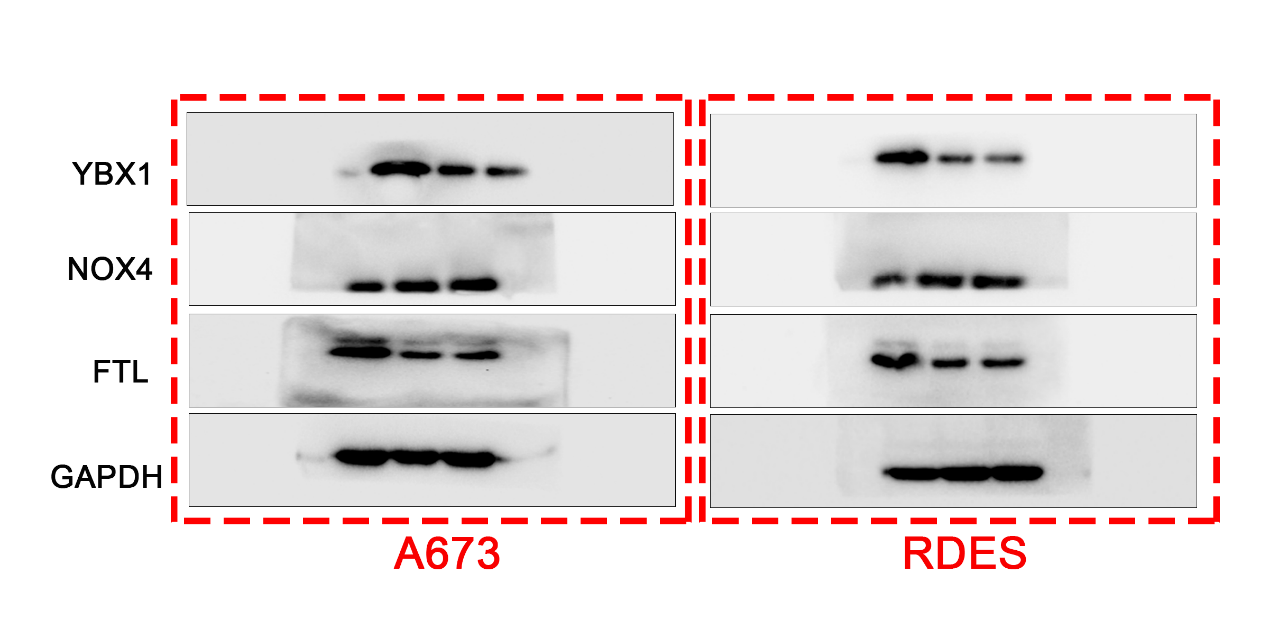
**

**Fig S16. Original full length western blots for Fig S6.**

**
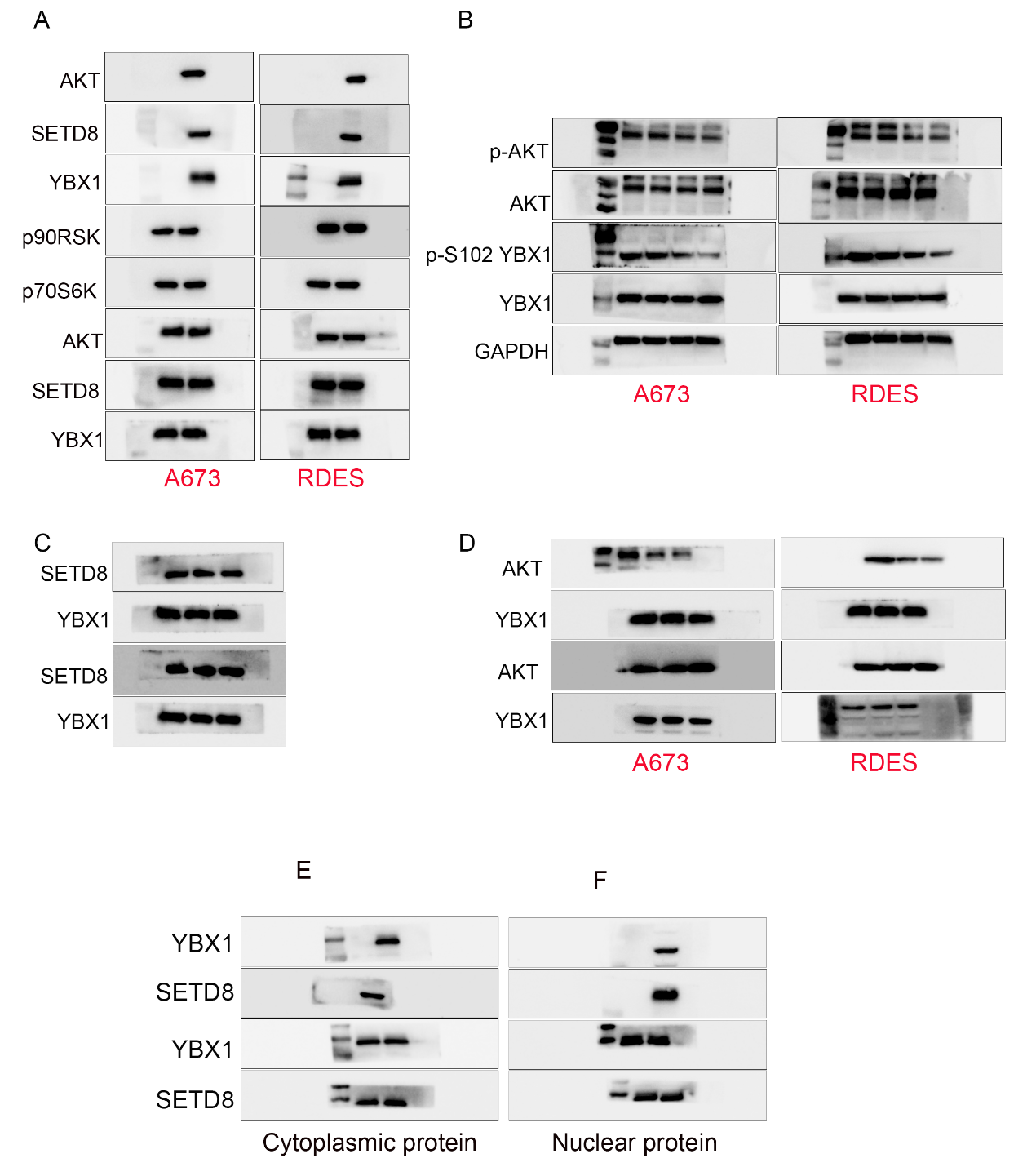
**

**Fig S17. Original full length western blots for Fig S7.**

**
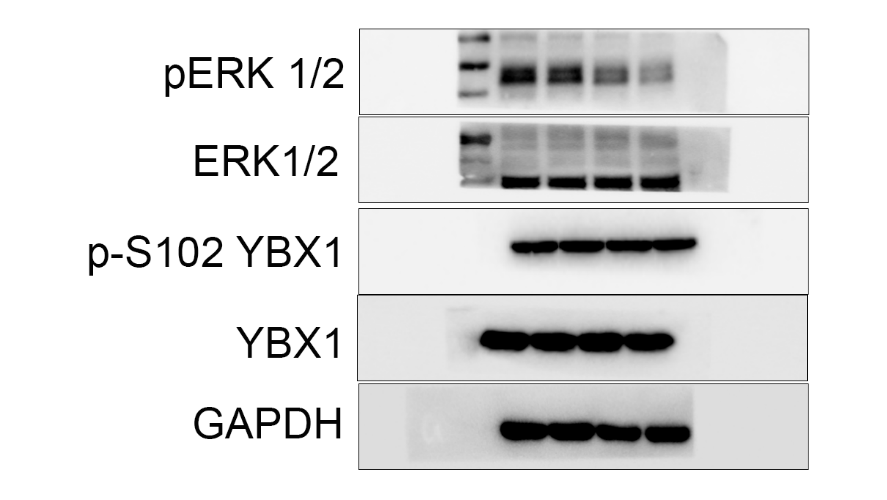
**

**Fig S18. Original full length western blots for Fig S9.**


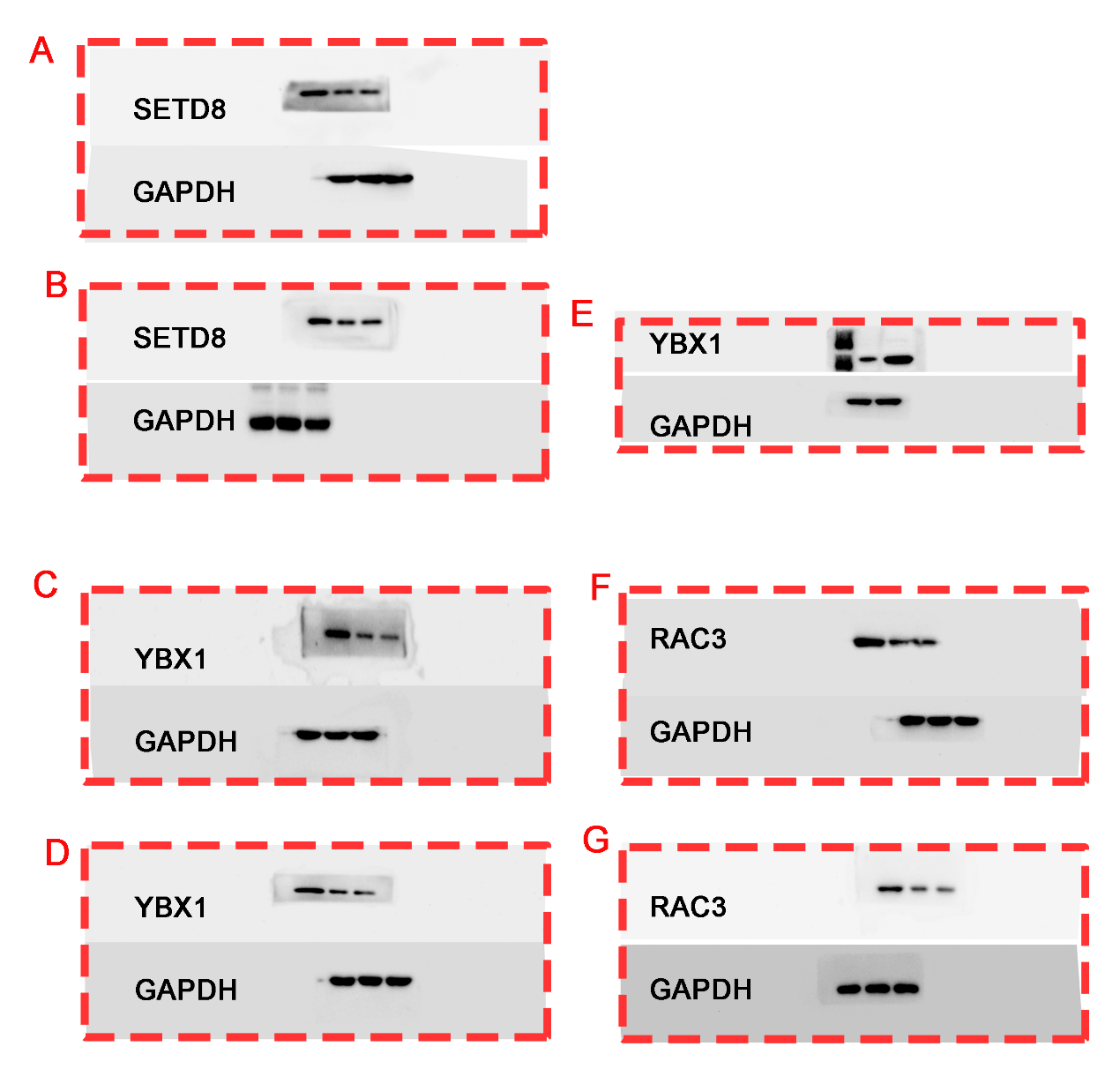

Supplement: Supplementary file 1 — supplementary material [file 41419_2024_6882_MOESM1_ESM.docx]
