# Supplementary material for: SETD8 inhibits apoptosis and ferroptosis of Ewing’s sarcoma through YBX1/RAC3 axis
Source: Cell Death Dis. 2024 Jul 10;15(7):494. doi: 10.1038/s41419-024-06882-5 (PMC11237091; doi:10.1038/s41419-024-06882-5)
Supplement: Supplementary file 2 — The sequence of primers used in this study [file 41419_2024_6882_MOESM2_ESM.docx]

| Name | Forward primer (5'->3') | Reverse primer (5'->3') |
| --- | --- | --- |
| GAPDH | TCGTCATGGGTGTGAACCAT | TGATGATCTTGAGGCTGTTGTCA |
| RAC3 | GGGAAGACATGCTTGCTGAT | CCGATCGTAGTCCTCCTGAC |
| YBX1 | TCCCAAAGTGCTGGGATTAC | ACTCCCGACCCCTACTGTCT |
| MAP2K6 | TCAATGCTCTCGGTCAAGTG | GTGATGCCCAGACTCCAAAT |
| RPS6KA2 | ACTGGCTCTCCTTCCTCACA | CCACTGCAGATTGGGAGAAT |

**Table S1 The sequence of primers used in this study**
